# Accelerometry-derived features of physical activity, sleep and circadian rhythm relate to non-motor symptoms in individuals with isolated REM sleep behavior disorder

**DOI:** 10.1007/s00415-025-12931-6

**Published:** 2025-02-11

**Authors:** Anja Ophey, Vaishali Vinod, Sinah Röttgen, Daniel Scharfenberg, Gereon R. Fink, Michael Sommerauer, Elke Kalbe, Walter Maetzler, Clint Hansen

**Affiliations:** 1 Medical Psychology | Neuropsychology and Gender Studies, Center for Neuropsychological Diagnostics and Intervention (CeNDI), Faculty of Medicine and University Hospital Cologne, University of Cologne, Cologne, Germany; 2 Cognitive Neuroscience, Institute for Neuroscience and Medicine, INM-3, Research Centre Juelich, Juelich, Germany; 3 Department of Neurology, University Medical Center Schleswig-Holstein Campus Kiel and Kiel University, Kiel, Germany; 4 Department of Neurology, Faculty of Medicine and University Hospital Cologne, University of Cologne, Cologne, Germany; 5 Center of Neurology, Department of Parkinson, Sleep and Movement Disorders, University of Bonn, Bonn, Germany

**Keywords:** Actigraphy, Wearables, Digital biomarkers, Digital health technology, Prodromal Parkinson’s disease

## Abstract

Accelerometry enables passive, continuous, high-frequency monitoring under free-living conditions. For individuals with isolated REM sleep behavior disorder (iRBD), a potential prodromal phase of Parkinson’s disease (PD) and dementia with Lewy bodies, accelerometry has been primarily applied to aid diagnosis and to assess phenoconversion risk. To extend this knowledge, we cross-sectionally combined clinical assessments focusing on non-motor symptoms with accelerometry-derived features of physical activity (PA), sleep, and circadian rhythm of *N* = 68 individuals with iRBD (age: 69.48 ± 6.01 years, self-reported RBD symptom duration: 9.46 ± 6.21 years, 85 % male). Accelerometry-assessed PA was associated with more stable circadian rhythms. Additionally, higher PA and more stable circadian rhythms were linked to a lower burden of overall non-motor symptoms, depressive symptoms, and fatigue with small to moderate effect sizes. Furthermore, including accelerometry-derived features improved the prediction of individual clinical scores, particularly for cognitive performance. Our findings contribute to the growing body of evidence highlighting the complex interplay between PA, sleep, circadian rhythm, and non-motor symptoms in α-synucleinopathies. Future research should focus on longitudinal studies to monitor changes in clinical outcomes and digital biomarkers over time to enhance our understanding of symptom progression and corresponding lifestyle changes in prodromal and manifest α-synucleinopathies.

**Supplementary Information:**

The online version contains supplementary material available at 10.1007/s00415-025-12931-6.

## Introduction

Isolated rapid eye movement (REM) sleep behavior disorder (iRBD) represents a non-REM parasomnia and may indicate an early α-synucleinopathy [1, 2] with most affected individuals developing clinically manifest Parkinson’s disease (PD) or dementia with Lewy bodies (DLB) within the next decade [3, 4]. In the years before the clinical diagnosis of PD or DLB, motor and non-motor symptoms, including cognitive, neuropsychiatric, and autonomic symptoms, progress [3, 5]. Mainly non-motor symptoms are strong predictors of quality of life in individuals with iRBD [6] and also for people with clinical PD [7, 8]. However, the monitoring of these symptoms via self-reports and rating scales during clinical visits poses multiple challenges, including the time and cost of in-person appointments, the inherent subjectivity of self-reports, a limited capability to capture symptom fluctuations, limited reliability, and a lack of ecological validity due to the artificial clinical setting [9, 10].

In PD, digital measures are already recognized as powerful tools to remotely monitor both motor and non-motor symptoms, potentially revolutionizing outcome assessments in future disease-modifying trials [10–12]. For example, features derived from wrist-worn accelerometry under free-living conditions may identify PD up to 7 years before the clinical diagnosis [13] and correlate with one of the gold-standard assessments of motor symptom severity, the motor part of the Unified Parkinson’s Disease Rating Scale, UPDRS-III [11]. Accelerometry non-invasively and passively collects raw data at potentially high sampling rates (e.g., 100 Hz) that can be processed to features allowing the study of physical activity, sleep, and circadian rhythm.

As recently reviewed [14], the potential of actigraphy is also increasingly recognized in the field of iRBD research. So far, it has been preferentially applied to aid the identification of iRBD [15–17]. Increasing levels of inactivity (i.e., napping behavior and time spent in inactivity) and activity fragmentation [18], as well as lower sleep efficiency and wake after sleep onset (WASO) [15] characterized individuals with iRBD compared to healthy controls (HC).

It has recently been demonstrated that physical activity and sleep measures collected by smartwatches significantly relate to clinical non-motor assessments of cognitive, autonomic, and daily living impairment in people with PD [19]. However, their predictive performance was poor on an individual level [19], underscoring the need to better understand what these digital outcomes actually reflect, as well as their relation to clinical symptoms assessed by the clinical gold standards. Further developments of this work should involve moving from the clinical to the prodromal phase of PD, as non-motor symptoms dominate this phase and precede the onset of PD-related motor symptoms by years [20, 21]. Additionally, using open-source algorithms to process raw accelerometry data, e.g., from the GGIR environment [22, 23], offers greater potential for reproducibility and refinement of the algorithms used.

With the present work, we aim (i) to explore the inherent relationship between accelerometry-derived measures of physical activity, sleep, and circadian rhythm in individuals with iRBD, (ii) to investigate the relationship of these accelerometry-derived measures with both motor and non-motor assessments (e.g., cognition, depressive symptoms, fatigue) in individuals with iRBD, and (iii) to evaluate the predictive potential of these accelerometry-derived measures for clinical outcomes in individuals with iRBD.

## Methods

### Study design and participants

This cross-sectional study utilized data assessed between 06/2022 and 09/2024 during the ongoing baseline assessments for the randomized controlled trial CogTrAiL-RBD [24] at the University Hospital Cologne in Germany. Participants were recruited from our local iRBD cohort [25] and HC via newsletters and flyers.

Individuals with iRBD fulfilled the following inclusion criteria: (i) diagnosis of iRBD confirmed by video-polysomnography, (ii) age between 40 and 80 years, (iii) normal or corrected-to-normal vision and hearing, and (iv) German as native tongue or sufficient proficiency in German. Exclusion criteria for individuals with iRBD were: (i) severe cognitive dysfunctions (Montréal Cognitive Assessment, MoCA, ≤ 22)[26], and (ii) significant neurological and psychiatric concomitant diseases. The same in- and exclusion criteria were applied for HC plus the absence of diagnoses of movement disorders, signs of iRBD, or any other psychiatric and neurological condition (including mild cognitive impairment, MCI, as assessed by Level-II cognitive assessment [27]). For the present analyses, we only included participants with valid data of the accelerometry module at baseline as specified under “Accelerometry Preprocessing”.

### Assessments

All participants in CogTrAiL-RBD attended clinical and neuropsychological assessments, while the accelerometry module was optional. After the in-person assessments, participants digitally completed questionnaires on various non-motor symptoms.

#### Clinical assessments: non-motor and motor symptoms

The presence of non-motor symptoms was assessed with the Movement Disorder Society (MDS) Non-Motor Symptoms Questionnaire (NMSQ) [28], fatigue with the Fatigue Scale for Motor and Cognitive Functions (FSMC) [29], and depressive symptoms with the Beck Depression Inventory (BDI-II) [30]. Subjective sleep quality was assessed with the Parkinson’s Disease Sleep Scale 2 (PDSS-2) [31] and RBD symptoms with the REM Sleep Behavior Disorder Screening Questionnaire (RBDSQ) [32]. For all these scales higher scores indicate more symptoms or a higher symptom severity, respectively. The Purdue Pegboard (dominant hand) was utilized to evaluate fine motor abilities [33], with obtained scores transformed to age- and sex-corrected *z*-scores using published normative data. PD-related motor impairment was evaluated with the MDS Unified Parkinson’s Disease Rating Scale (MDS-UPDRS-III, higher scores indicate more motor impairment) [34]. The MDS-UPDRS-III was recorded on video during the baseline visit and scored by neurologists with expertise in movement disorders blinded for group (HC vs. iRBD). In addition to the MDS-UPDRS-III total score, tremor, rigidity, axial, and bradykinesia subscores were calculated.

#### Cognitive assessments

The MoCA [26] was applied as a cognitive screening. A Level-II cognitive test battery including at least two tests for each of the five primary cognitive domains (executive functions, attention and working memory, memory, visuo-cognition, language) was applied following the MDS guidelines for the operationalization of MCI in PD [27]. Test scores were demographically adjusted and standardized using published normative data and transformed into *z*-scores during data preprocessing (higher scores indicate better cognitive performance). An overview of the assignment of cognitive tests to the respective domains and details on the applied MCI criteria is presented in the Online Resource Table S1. Equally weighted cognitive domain composite scores were calculated as the mean *z*-score of tests within one cognitive domain. Furthermore, an equally weighted global cognition composite score was calculated based on the cognitive domain composite scores. Besides the standard cognitive test battery, the Timed Up and Go Test in a dual-task condition (counting backwards in steps of 7) was conducted as a measure for cognitive-motor dual-tasking [35].

#### Accelerometry

Physical activity and circadian rhythm were measured with the AX6 device (Axivity Ltd, Newcastle upon Tyne, England), a 6-axis movement sensor equipped to measure 3D acceleration and 3D angular velocity. Participants were instructed to continuously wear the device on their dominant wrist for 7 days and nights (168 h) following the baseline assessment. The accelerometry sensor collected data at a sampling rate of 100 Hz with a range of ± 8 *g* (gyroscope disabled).

### Accelerometry preprocessing

**Fig. 1:**
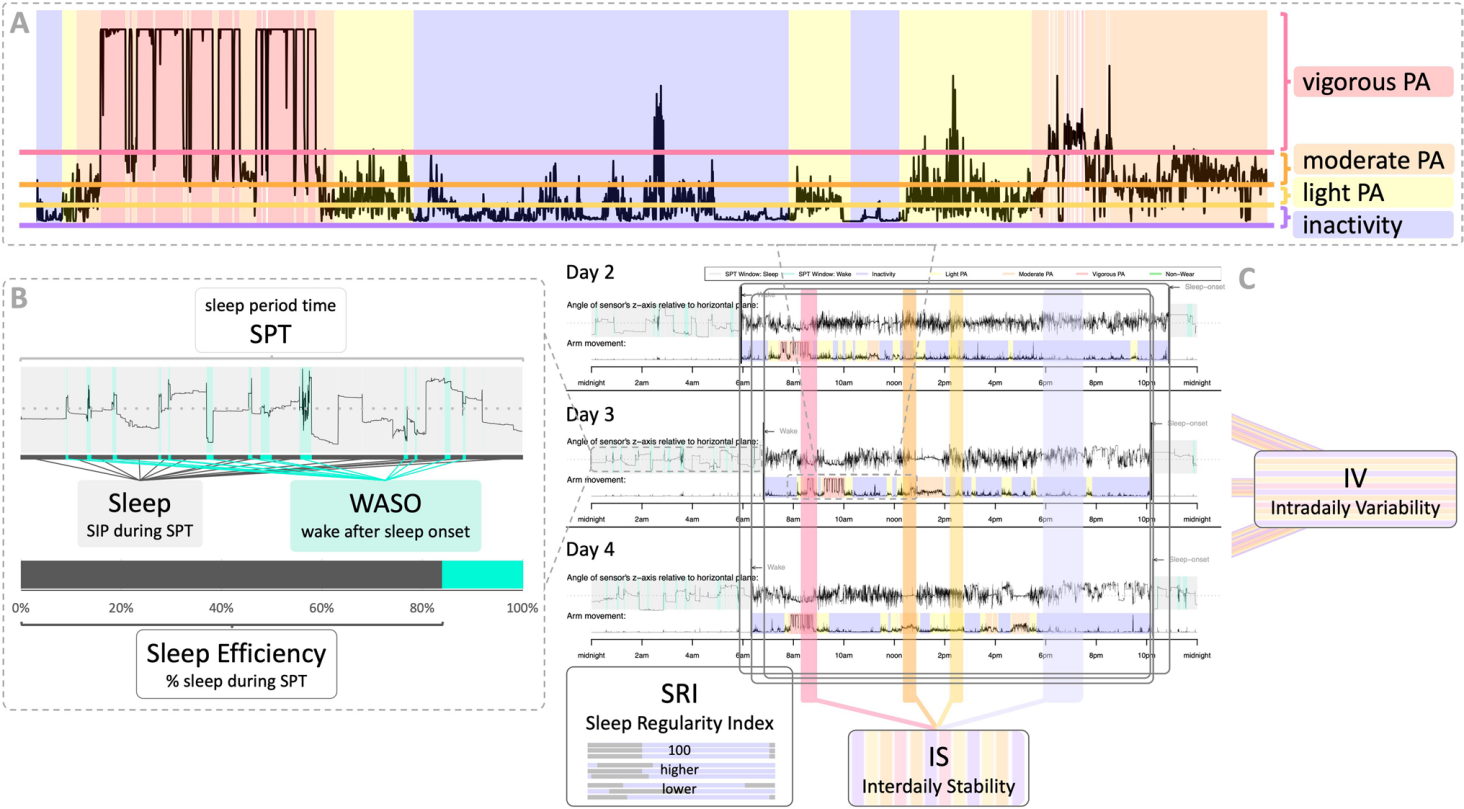
Illustration of actigraphy-derived measures for physical activity, sleep, and circadian rhythm generated by GGIR. The illustration is based on screenshots from the visual report generated by GGIR [22] (version 3.1.4). **A** Zoom on the physical activity summary of the overall arm movement during wake periods utilizing the default Euclidean norm minus one (ENMO) gravitational unit thresholds for inactivity (lilac), light physical activity (PA, yellow), moderate PA (orange), and vigorous PA (pink). **B** Zoom on the sleep summary. The sleep duration (dark grey) was measured as the total time of sustained inactivity periods (SIP) [38] within the sleep period time (SPT) [39] window, allowing us to compute sleep efficiency as the percentage of time of SIP within the SPT window. Wake after sleep onset (turquoise) is identified by changes in the z-axis of more than 5°. **C** Screenshot of three exemplary days of the visual report. The Sleep Regularity Index (SRI) reflects the probability of an individual being in the same state (asleep = grey vs. awake = lilac) at any two time points 24 h apart [40]. The higher the score, the more regular the sleep–wake cycles are throughout the time period. Intradaily variability (IV) [41] represents within-day fragmentation in the circadian rest-activity rhythm with lower values indicating a less fragmented rhythm. Interdaily stability (IS) [41] represents between-day rhythm consistency with higher values indicating a more stable rhythm across days

Raw accelerometer data were preprocessed in R [36] with the open-source GGIR [22] R-package (version 3.1.4), which was designed to process multi-day raw accelerometer data for physical activity, sleep, and circadian rhythm research. Only subjects with at least 5 days with ≥ 16 h/day of data were considered valid and used for analysis [22]. All valid measurements were used to analyze physical activity and valid measurements of at least two consecutive days were used for analysis of sleep and circadian rhythm variables. Remaining data were visually inspected for quality control as recommended by GGIR. During pre-processing, GGIR’s default auto-calibration [37] was applied. Calibration errors pre and post auto-calibration, the number of 10 s epochs used as sphere data during auto-calibration, the clipping score, and the non-wear percentage during the whole day are reported in Online Resource Table S2. Figure 1 presents a screenshot and zoom-ins of an exemplary visual summary output provided by GGIR (initially developed to show a report to participants), illustrating the actigraphy-derived features of interest for physical activity, sleep, and circadian rhythm extracted for the present study (for an overview and specific GGIR variable names, see Online Resource Table S2).

The GGIR cut points to classify the intensity of physical activity are based on the Euclidean norm minus one (ENMO) gravitational unit (*g*) [42]. *Total-ENMO*, derived from the raw accelerometer data, provides a single numerical value representing the overall magnitude of acceleration in all axes per day (i.e., between midnight and midnight) averaged across all days. For the time spent in specific intensity levels of physical activity during wake time (i.e., time outside the sleep period time, SPT, window as described below), the intensity levels of physical activity were categorized based on the default ENMO thresholds in GGIR [22], i.e., 0–39 m*g* corresponding to *inactivity*, 40–99 m*g* to *light physical activity (LPA)*, 100–399 m*g* to *moderate physical activity (MPA)*, and ≥ 400 m*g* to *vigorous physical activity (VPA)*.

Concerning sleep, GGIR estimates the SPT based on the Heuristic algorithm looking at the Distribution of Change in Z-Angle (HDCZA) [39], enabling the detection of the SPT without guidance by a sleep diary. The algorithm identifies sustained inactivity periods (SIP) as the absence of change in the Z-axis angle larger than 5° for more than 5 min [38] and makes basic assumptions about sleep interruptions [39]. The sleep duration was measured as the total time of SIP within the SPT window, allowing us to compute *sleep efficiency* as the percentage of time of SIP within the SPT window. Sleep continuity was quantified by the time spent awake during the SPT (*WASO, wake after sleep onset*).

The *Sleep Regularity Index (SRI)* reflects the probability of an individual being in the same state (asleep vs. awake) at any two time points 24 h apart [40]. The higher the score, the more regular the sleep–wake cycles are throughout the time period. *Interdaily stability (IS)* and *intradaily variability (IV)* were calculated as implemented in GGIR according to the non-parametric approach of van Someren, Hagebeuk [41]. IV represents within-day fragmentation in the circadian rest-activity rhythm with lower values indicating a less fragmented rhythm. IS represents between-day rhythm consistency with higher values indicating a more stable rhythm across days.

### Statistical analyses

Data were analyzed using R [36] with *ggplot2* (version 3.5.0) and *corrplot* (version 0.92) for data visualization. Demographic, clinical and cognitive variables as well as the GGIR-derived features on physical activity, sleep, and circadian rhythm were compared between HC and individuals with iRBD using non-parametric Mann–Whitney *U-*tests.

Partial Spearman’s correlation coefficients (ρ) controlling for age were computed to test the association between the GGIR-derived features on physical activity, sleep, and circadian rhythm, and to test the association between these features and the clinical scores of cognition, and motor- and non-motor symptoms in the iRBD sample only. The significance level was adjusted using the Benjamini–Hochberg procedure controlling the false discovery rate (FDR) across multiple comparisons. Additionally, partial Pearson correlation analyses were conducted to ensure that we do not miss associations potentially driven by boundary or extreme values.

Two models per clinical outcome were compared to evaluate the added value of digital accelerometry-derived features beyond basic demographic characteristics and iRBD symptom duration to predict individual clinical scores. A regression model using the digital features of physical activity, sleep, and circadian rhythm as predictors, with age, sex, and time since first RBD symptoms as covariates was built as the ‘full model’. This full model was compared to a model based on covariates only (‘base model’) for each clinical score. Predictors and covariates were mean-centered prior to model estimation. All models employed elastic net regularization and were fitted using a nested ten-fold cross-validation. In the inner folds, a grid search was conducted to optimize hyperparameters, specifically the alpha (L1 to L2 ratio) and lambda (penalty strength). Model performance was reported as the mean and standard deviation of the *R*^2^ scores across the ten outer folds and compared between the base and full models with two-sided independent sample *t*-tests.

## Results

### Sample characteristics

Of 72 individuals with iRBD included in the CogTrAiL-RBD study until 09/2024, *n* = 70 agreed to the accelerometry module (97.2% acceptance rate). Out of the 70 collected data sets, 2 (2.9%) were invalid (insufficient wear time), resulting in a total of *n* = 68 data sets with ≥ 5 days of valid data. For HC, *n* = 24 valid data sets were available for the present analyses. Of initially *n* = 26 HC AX6 assessments, *n* = 2 (7.7%) data sets had to be excluded due to insufficient wear time.

Individuals with iRBD were 69.48 ± 6.01 years old, reported a time of 9.46 ± 6.21 years since their first RBD symptoms and *n* = 58 (85%) were male. Individuals with iRBD and HC were comparable regarding demographic characteristics, including age, sex distribution, education and occupational status (Table 1). Twenty (29%) individuals with iRBD fulfilled the Level-II diagnostic criteria for MCI; however, neither MoCA scores nor the cognitive domain composite scores significantly differed between iRBD and HC (*p*_FDR_ > 0.05). Compared to HC, individuals with iRBD performed worse in the Purdue Pegboard (dominant hand, *U* = 1158, *p*_FDR_ = 0.007, |*r*|= 0.32). They reported more non-motor symptoms (NMSQ, *U* = 246, *p*_FDR_ < 0.001, |*r*|= 0.51), fatigue (FSMC, *U* = 408, *p*_FDR_ = 0.004, |*r*|= 0.35), depressive symptoms (BDI-II, *U* = 333, *p*_FDR_ < 0.001, |*r*|= 0.43), and sleep disturbances (PDSS, *U* = 489, *p*_FDR_ = 0.035, |*r*|= 0.27; RBDSQ, *U* = 58.5, *p*_FDR_ < 0.001, |*r*|= 0.7). Further details on clinical characteristics, comorbidities, and medication can be found in Online Resource Table 3.

**Table 1:** Sample Characteristics

		Healthy Controls *N* = 24	iRBD *N* = 68	*p*_FDR_
Age *in years*		67.96 (4.33) [61.16 – 77.97]	69.48 (6.01) [55.90 – 80.93]	0.386
sex, *n (%)*	male	21 (87.50 %)	58 (85.29 %)	> 0.999
	female	3 (12.50 %)	10 (14.71 %)	
Time since first reported RBD symptoms *in years*			9.46 (6.21) [1.92 – 30.93]	
RBD-related medication		0 (0 %)	7 (10.29 %) *Circadin n* = *1* *Clonazepam n* = *1* *Melatonin n* = *5*	
Education *in years*		17.33 (3.79) [11.50 – 27]	15.68 (3.03) [7 – 22]	0.249
Occupational status	employed / self-employed	8 (33.33 %)	12 (17.65 %)	0.404
	not employed / on leave	0 (0 %)	3 (4.41 %)	
	retired	16 (66.67 %)	53 (77.94 %)	
MCI status, *n (%)*	noMCI	24 (100.00 %)	48 (70.59 %)	0.007
	MCI	0 (0.00 %)	20 (29.41 %)	
MoCA [0–30]		26.88 (2.13) [23 – 30]	26.18 (2.15) [23 – 30]	0.386
Global Cognition *z-score*		0.46 (0.21) [0.15 – 0.97]	0.38 (0.35) [−0.44 – 1.23]	0.420
Executive Functions *z-score*		0.43 (0.34) [−0.14 – 1.19]	0.36 (0.49) [−1.19 – 1.25]	0.891
Attention & Working Memory *z-score*		0.66 (0.56) [−0.25 – 1.75]	0.50 (0.58) [−0.60 – 1.99]	0.420
Memory *z-score*		0.39 (0.67) [−1.13 – 1.50]	0.29 (0.63) [−1.21 – 1.68]	0.631
Visuo-Cognition *z-score*		0.55 (0.30) [−0.25 – 1.05]	0.55 (0.38) [−0.30 – 1.37]	0.910
Language *z-score*		0.28 (0.27) [−0.25 – 0.71]	0.20 (0.34) [−0.49 – 1.06]	0.503
Timed Up and Go Cost *in seconds*		−2.17 (1.67) [−6.00 – 0.00]	−2.91 (3.12) [−19.00 – 2.00]	0.600
Purdue Pegboard, dominant hand		−0.13 (0.88) [−1.93 – 1.38]	−0.79 (0.88) [−3.25 – 1.15]	0.007
MDS-UPDRS-III* [0–132]		4.11 (4.01) [0 – 14]	5.20 (4.52) [0 – 19]	0.495
MDS-UPDRS-III tremor* [0–40]		0.39 (0.92) [0 – 3]	0.22 (0.73) [0 – 4]	0.600
MDS-UPDRS-III rigidity* [0–20]		0.88 (0.90) [0 – 3]	2.54 (1.57) [0 – 8]	< 0.001
MDS-UPDRS-III axial* [0–24]		0.39 (0.85) [0 – 3]	0.92 (1.09) [0 – 5]	0.077
MDS-UPDRS-III bradykinesia* [0–32]		2.72 (3.08) [0 – 10]	3.26 (3.14) [0 – 14]	0.503
PASE^#^		133.03 (53.78) [58.00 – 253.21]	137.81 (67.41) [42.25 – 354.36]	0.993
NMSQ^#^ [0–30]		2.58 (1.98) [0 – 8]	6.77 (3.83) [0 – 18]	< 0.001
FSMC^#^ [20–100]		26.63 (5.76) [20–42]	38.63 (15.57) [20 – 88]	0.004
BDI-II^#^ [0–63]		1.33 (1.58) [0 – 6]	6.41 (6.33) [0 – 28]	< 0.001
PDSS-2^#^ [0–60]		9.58 (3.26) [4 – 16]	12.59 (4.93) [6 – 29]	0.028
RBDSQ^#^ [0–13]		2.08 (1.56) [0 – 5]	8.66 (2.80) [0 – 13]	< 0.001

Data are mean (standard deviation) [Range: minimum – maximum] unless indicated otherwise. Healthy controls and individuals with iRBD were compared with Mann–Whitney U-tests for continuous variables and *χ*^2^-tests for categorical variables

BDI-II, Beck Depression Inventory; FSMC, Fatigue Scale for Motor and Cognitive Functions; iRBD, isolated REM sleep behavior disorder; MCI, mild cognitive impairment; MDS-UPDRS-III, Movement Disorder Society Unified Parkinson’s Disease Rating Scale Part 3; MoCA, Montréal Cognitive Assessment; NMSQ, Non-Motor Symptom Questionnaire; PASE, Physical Activity Scale for the Elderly, PDSS, Parkinson’s Disease Sleep Scale; RBDSQ, REM Sleep Behavior Disorder Screening Questionnaire.

*n_HC_ = 16, *n*_RBD_ = 46

^#^*n*_HC_ = 21, *n*_RBD_ = 64

**Fig. 2:**
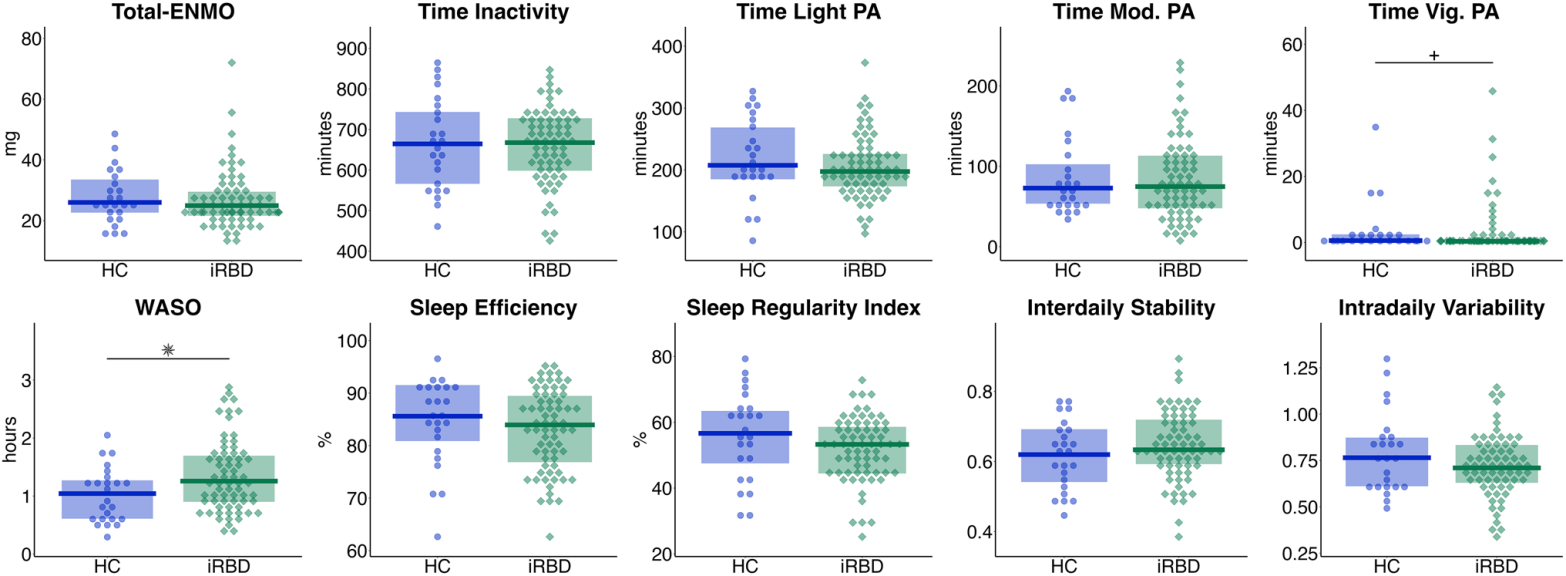
Actigraphy-derived measures for physical activity, sleep, and circadian rhythm in iRBD compared to HC**.** For the domains of physical activity (PA), sleep, and circadian rhythm, dots (HC, blue) and squares (iRBD, green) represent individual scores in the variables of interest, the group-wise boxplots visualize the within-group median, the hinges represent the corresponding first and the third quartile. ENMO, Euclidean norm minus one gravitational unit; HC, healthy controls; iRBD, isolated REM sleep behavior disorder; Mod., moderate; PA, physical activity; Vig., vigorous; WASO, wake after sleep onset. * *p* < 0.05, ^+^ *p* < 0.10, uncorrected

Total-ENMO did not differ between HC (*M* = 27.58, *SD* = 8.69) and individuals with iRBD (*M* = 26.86, *SD* = 9.76), *U* = 893, *p*_FDR_ = 0.617, |*r*|= 0.07. Regarding the digital measures of physical activity, sleep, and circadian rhythm (Fig. 2), there was more WASO in iRBD compared to HC with a small effect size (*U* = 557, *p* = 0.021, |*r*|= 0.24), however, significance did not survive FDR-correction (*p*_FDR_ = 0.180). There were no statistically significant differences in variables relating to daytime physical activity (i.e., time spent in inactivity, LPA, MPA, VPA), sleep efficiency and circadian rhythm (SRI, IS, IV). For details, see Online Resource Table S3

### Relationship between accelerometry-derived physical activity, sleep, and circadian rhythm features in iRBD

**Fig. 3:**
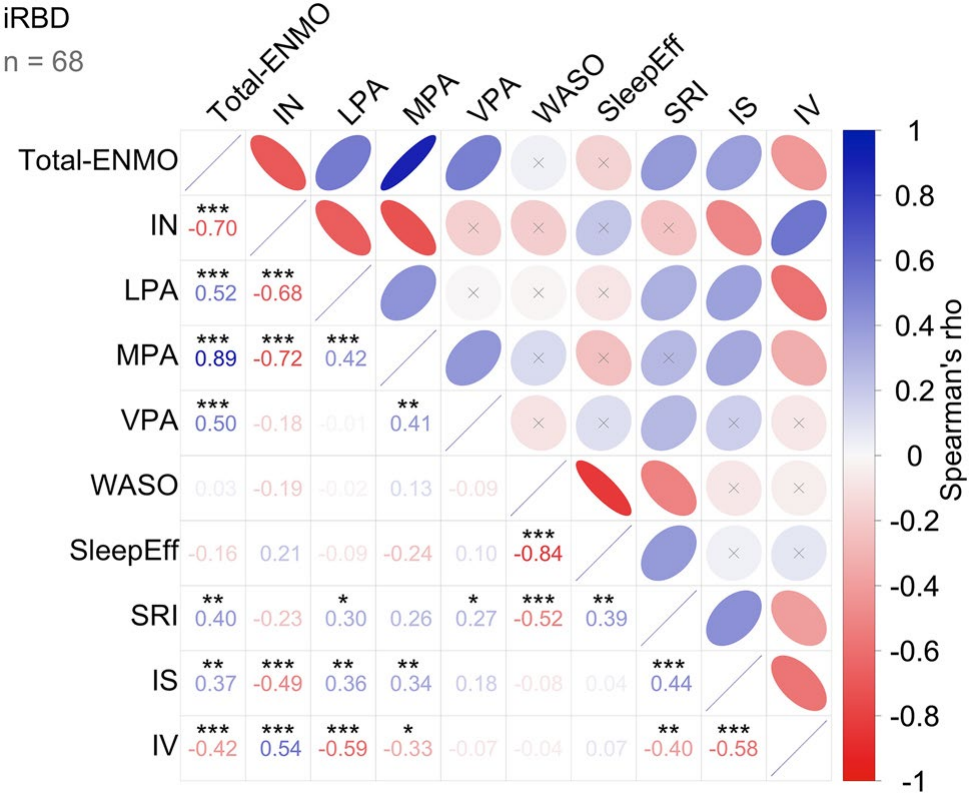
Relationship between Physical Activity, Sleep, and Circadian Rhythm in iRBD. The correlation matrix presents the partial Spearman’s rank correlations adjusted for age. Ellipses in the upper triangle visualize the strength of the observed correlations (narrower ellipses indicate stronger correlations). In the lower triangle, Spearman’s ρ and the corresponding FDR-corrected significance levels are displayed. For details, see Online Resource Table S4. IN, time spent in inactivity; iRBD, isolated REM sleep behavior disorder; IS, interdaily stability; IV, intradaily variability; LPA, time spent in light physical activity; MPA, time spent in moderate physical activity; SleepEff, sleep efficiency; SRI, Sleep Regularity Index; Total-ENMO, Euclidean norm minus one (ENMO) gravitational unit; VPA, time spent in vigorous physical activity; WASO, wake after sleep onset. *** *p*_FDR_ < 0.001, ** *p*_FDR_ < 0.01, * *p*_FDR_ < 0.05.

Figure 3 provides a visualization of the Spearman correlation matrix with FDR-corrected significance levels (for details, see Online Resource Table S4). Total-ENMO, the overall acceleration, correlated strongly positive with time spent in all activity levels (LPA, MPA, VPA), and strongly negative with time spent in inactivity. No meaningful association existed between the activity variables and sleep continuity (WASO) or sleep efficiency. The SRI showed a moderate positive correlation with Total-ENMO, time spent in LPA and VPA, and Sleep Efficiency, indicating that more sleep regularity was related to more physical activity and higher sleep efficiency. Additionally, the SRI showed a strong negative association with WASO, revealing less sleep regularity to be related to less sleep continuity. The correlations with time spent in inactivity (negative), and MPA (positive) were weak and failed the FDR-corrected significance threshold. IS correlated moderately positive with physical activity (Total-ENMO, LPA, MPA) and the SRI. Its association with time spent in inactivity was strongly negative. The opposite associations were observed for IV. These findings indicate a more stable circadian rhythm and less within-day variability to be linked to more physical activity and higher sleep regularity. The results of the Pearson correlation analysis are reported in Online Resource Table S5.

### Relationship of accelerometry-derived features and clinical scores in iRBD

**Fig. 4:**
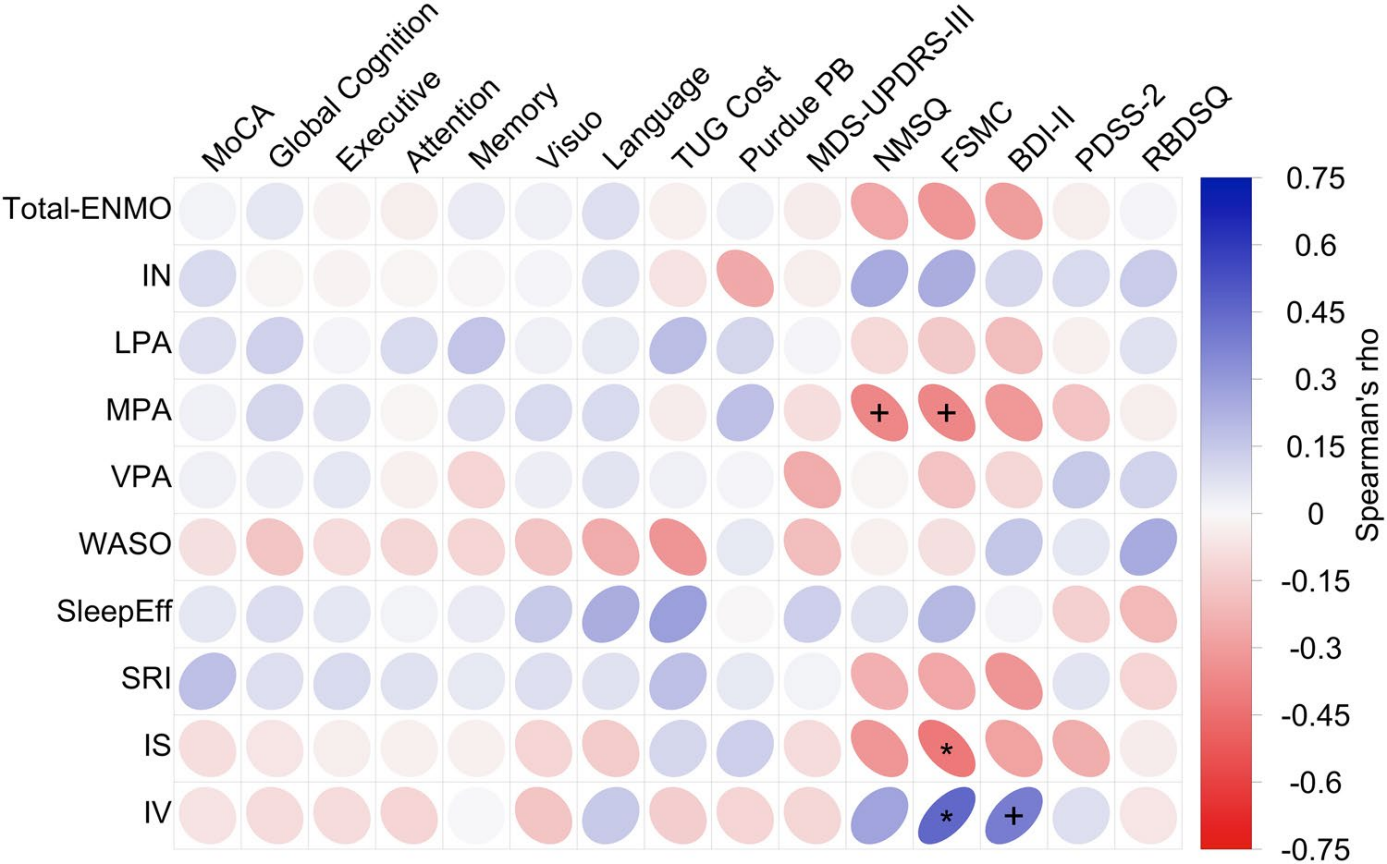
Relationship of Accelerometry-Derived Measures and Clinical Assessments in iRBD. The correlation matrix reports the partial Spearman’s rank correlations adjusted for age. Ellipses visualize the strength of observed correlations (the narrower the stronger). For details, see Online Resource Table S6. BDI-II, Beck Depression Inventory; FSMC, Fatigue Scale for Motor and Cognitive Functions; IN, time spent in inactivity; iRBD, isolated REM sleep behavior disorder; IS, interdaily stability; IV, intradaily variability; LPA, time spent in light physical activity; MDS-UPDRS-III, Movement Disorder Society Unified Parkinson’s Disease Rating Scale Part 3; MoCA, Montréal Cognitive Assessment; MPA, time spent in moderate physical activity; NMSQ, Non-Motor Symptom Questionnaire; PDSS, Parkinson’s Disease Sleep Scale; Purdue PB, Purdue Pegboard dominant hand; RBDSQ, REM Sleep Behavior Disorder Screening Questionnaire; SleepEff, sleep efficiency; SRI, Sleep Regularity Index; Total-ENMO, Euclidean norm minus one (ENMO) gravitational unit; TUG Cost, Timed Up and Go Cost single task—dual task; VPA, time spent in vigorous physical activity; WASO, wake after sleep onset

Figure 4 provides a visualization of the Spearman correlation matrix with FDR-corrected significance levels (for details, Online Resource Table S6). Overall, correlations between the accelerometry-derived features and clinical outcomes in individuals with iRBD were weak to moderate. Non-motor symptoms (NMSQ, FSMC, BDI-II) consistently showed negative weak to moderate associations with physical activity, indicating that higher physical activity tended to be linked to fewer non-motor symptoms. This was particularly evident for time spent in MPA concerning the overall non-motor symptom burden (NMSQ, ρ(64) = −0.37, *p*_FDR_ = 0.081) and fatigue (FSMC, ρ(64) = − 0.37, *p*_FDR_ = 0.081). Similarly, this group of symptoms was negatively correlated with SRI and IS and positively with IV, revealing more stable circadian rhythms (higher SRI, higher IS, lower IV) to be linked to fewer non-motor symptoms. Their significance did mostly not survive FDR-correction, except for a negative association between IS and FSMC scores (ρ(64) = − 0.42, *p*_FDR_ = 0.045) and a positive association between IV and FSMC scores (ρ(64) = 0.45, *p*_FDR_ = 0.030), indicating that higher IS and lower IV were associated with less fatigue. Furthermore, a lower IV tended to be associated with less depressive symptoms (BDI-II, ρ(64) = 0.39, *p*_FDR_ = 0.075). The results of the Pearson correlation analysis are reported in Online Resource Table S7.

*** *p*_FDR_ < 0.001, ** *p*_FDR_ < 0.01, * *p*_FDR_ < 0.05, + *p*_FDR_ < 0.10.

### Prediction of clinical scores from accelerometry-derived measures

**Fig. 5:**
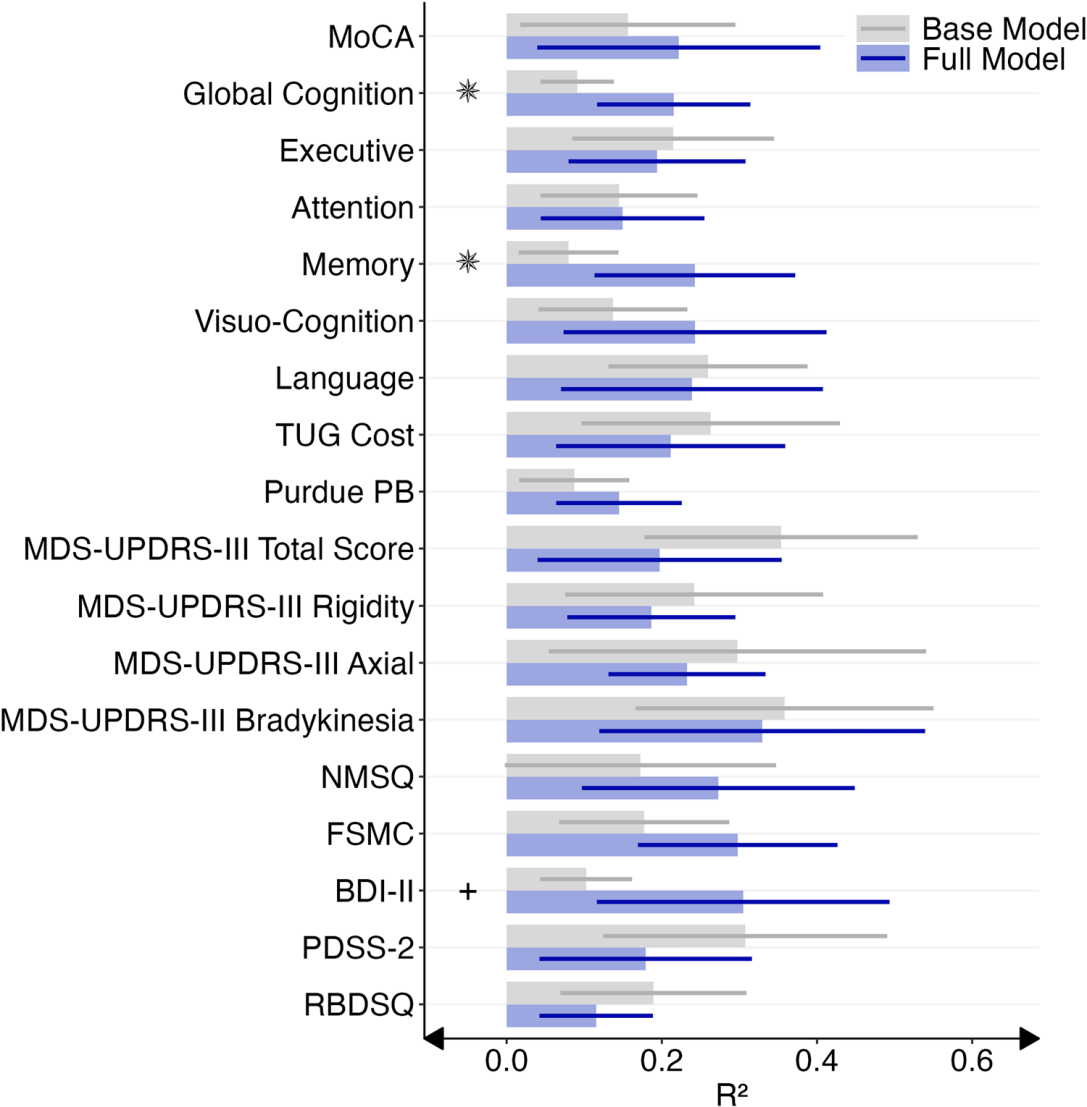
Prediction of Clinical Scores from Accelerometry-Derived Measures. The amount of explained variance (*R*^2^, x-axis) for each clinical measure (y-axis) is plotted as the mean *R*^2^ across the ten outer cross-validation folds with its 95 % confidence interval for full models (blue) based on accelerometry derived-features on physical activity, sleep, and circadian rhythm as predictors, and age, sex, and RBD symptom duration as covariates compared to base models (grey) based on the covariates only. An asterisk indicates a significant difference between the two models (two-sided *t*-test with *N* = 10 each). BDI-II, Beck Depression Inventory; FSMC, Fatigue Scale for Motor and Cognitive Functions; iRBD, isolated REM sleep behavior disorder; MDS-UPDRS-III, Movement Disorder Society Unified Parkinson’s Disease Rating Scale Part 3; MoCA, Montréal Cognitive Assessment; NMSQ, Non-Motor Symptom Questionnaire; PDSS, Parkinson’s Disease Sleep Scale; Purdue PB, Purdue Pegboard dominant hand; RBDSQ, REM Sleep Behavior Disorder Screening Questionnaire; TUG Cost, Timed Up and Go Cost single task—dual task. * *p* < 0.05, + *p* < 0.10, uncorrected.

The amount of explained variance (*R*^2^) for clinical outcomes ranged between 0.12 [RBDSQ, 95 % CI −0.04; 0.32] and 0.33 [MDS-UPDRS-III bradykinesia subscore, 95 % CI 0.12; 0.54] for full models based on accelerometry-derived features on physical activity, sleep, and circadian rhythm as predictors and age, sex and RBD symptom duration as covariates (Fig. 5, Online Resource Table S8). For base models based on the covariates only, mean *R*^2^ ranged between 0.08 [memory, 95 % CI 0.02; 0.14] and 0.36 [MDS-UPDRS-III bradykinesia subscore, 95 % CI 0.17; 0.55]. Only for the global cognition composite score (*t*(18) = −2.23, *p* = 0.039, Cohen’s *d* = −1.00) and the memory composite score (*t*(18) = −2.21, *p* = 0.040, Cohen’s *d* = −0.99), the full models performed significantly better than the base models. Descriptively, the full models outperformed the base models for most cognitive outcomes (except for executive functions and language) and non-motor symptoms (NMSQ, FSMC, BDI-II). Of note, the prediction of PD-related motor symptoms (MDS-UPDRS-III total score, and rigidity, axial and bradykinesia subscores), cognitive-motor dual tasking (TUG Cost) and sleep disturbances (PDSS, RBDSQ) did not benefit from the inclusion of accelerometry-derived features. For these outcomes, the base models descriptively outperformed the full models. Due to the extreme right skewness of the MDS-UPDRS-III tremor subscore (e.g., 89% of individuals with iRBD scored “0”), the corresponding models produced unreliable results and are not reported. Sensitivity analyses comparing the prognostic value of daytime vs. nighttime accelerometry-derived features are reported in Online Resource S9.

## Discussion

Our study demonstrates the relationship among accelerometry-derived features of physical activity, sleep, and circadian rhythm and their associations primarily with non-motor symptoms in individuals with iRBD, a potential prodromal phase of PD and DLB. Our key findings reveal (i) more physical activity to be associated with more stable circadian rhythms in iRBD, and (ii) weak to moderate correlations between accelerometry-derived features and clinical outcomes, that tend to be strongest for non-motor symptoms. More physical activity and more stable circadian rhythms were associated with less non-motor symptoms including overall non-motor symptom burden, depressive symptoms, and fatigue. This is underscored by our findings regarding the predictive potential of accelerometry-derived measures, which demonstrate that (iii) the inclusion of accelerometry-derived measures improved prediction of clinical outcomes primarily for non-motor outcomes, particularly global cognition and memory.

Within our sample, features of physical activity were similar between individuals with iRBD and HC, including overall Total-ENMO and the time spent in all activity levels (IN, LPA, MPA, VPA). Therefore, we could not replicate differences between individuals with iRBD and HC with regard to altered 24-h rest-activity measures [43] and sedentary behavior, the latter of which has been identified as a potential predictor for phenoconversion of individuals with iRBD to manifest PD [18]. Schalkamp, Peall [13] recently showed a reduced acceleration profile for people with prodromal and clinically diagnosed PD compared to a matched HC group. Thus, this finding may not generalize to individuals with iRBD. Individuals with iRBD are hypothesized to represent the body-first subtype of PD [44, 45], which is characterized by predominant non-motor symptoms (including iRBD, autonomic and neuropsychiatric symptoms, cognitive impairment) prior to the onset of parkinsonism. If developing parkinsonism would be the driving force for reduced physical activity in the prodromal phase, this could explain, why altered levels of physical activity were not strongly evident for individuals with iRBD. However, our results may also be biased with regard to lifestyle behaviors of our iRBD sample: Individuals with iRBD were volunteers of the local iRBD cohort participating in a clinical trial on cognitive training and promoting a healthy lifestyle [24]. Even though we only considered baseline data prior to randomization and any intervention, the nature of this trial may have attracted people who are particularly interested and motivated to pursue (or are already pursuing) a healthy, active lifestyle.

Furthermore, despite the commonly observed diagnostic delay in RBD [46], individuals in our iRBD group were not newly diagnosed cases. Considering typical phenoconversion rates following the iRBD diagnosis [4], our sample may therefore be biased towards more benign iRBD subtypes [47]. Conversely, the UK Biobank ‘prodromal cases’ may be biased towards more advanced prodromal stages, potentially including individuals with motor symptoms advanced enough to warrant a clinical PD diagnosis. This bias arises from the reliance on participants’ self-reported diagnoses in the UK Biobank, rather than on longitudinal, structured clinician assessments. Future replications of these findings in population-based samples and multi-centric projects on accelerometry as a digital biomarker in iRBD are warranted to evaluate the validity of using daytime activity measures for identifying iRBD. Daytime activity measures may be more valuable for understanding symptoms and tracking symptom progression within an iRBD sample, rather than for screening or diagnosing iRBD.

Our finding of a tendency for less sleep continuity regarding WASO aligns with previous accelerometry-based findings in iRBD [15]. However, accelerometry-derived WASO may be inherently linked to the nature of RBD behavior and increased WASO may not be specific to iRBD in comparison to other clinical (sleep) syndromes [15, 48]. More refined features tailored to RBD-specific behaviors, combined with established iRBD screening tools, have recently demonstrated near-perfect classification accuracy, underscoring the enormous potential of accelerometry for large-scale screening of iRBD in the general population [17]. In the future, implementing the underlying algorithms into existing toolboxes would enable broader applicability.

We found moderate to strong associations between the accelerometry-derived features of physical activity and circadian rhythm, replicating findings in multiple sclerosis and adopting a similar analysis within the GGIR environment [48], particularly about circadian rhythm regularity. Accelerometry-derived higher sleep regularity was associated with more time spent in activity and less inactivity during wake time. Sleep regularity is considered an essential contributor to overall health, including cardiovascular, mental, and cognitive health outcomes, as well as sleep quality and efficiency [49]. Physical activity may also impact the beneficial role of sleep regularity for overall health [50].

In fact, circadian stability seems to play a vital role for individuals with iRBD: greater sleep regularity and higher between- and within-day stability (higher IS, lower IV) were consistently associated with a lower symptom burden in the area of non-motor symptoms (overall non-motor symptom burden, fatigue, depression). The tendency for the observed association between increased physical activity and a reduced burden of non-motor symptoms in iRBD aligns with various findings from the field of PD research. Overall, data from the UK biobank revealed that more physical activity relates to a reduced risk of developing PD [51]. In a recent meta-analysis, more physical activity performed under free-living conditions, e.g., assessed by accelerometry, was associated with less affective disorders (depression, anxiety, apathy) and better global cognition for people with PD [52]. Cognitive outcomes were only marginally associated with single accelerometry-derived features on physical activity, sleep and circadian rhythm; however, the multiple regression models revealed the prognostic benefit of these features particularly for cognitive outcomes, underlining the complex interplay the domains under investigation.

Despite these consistent weak to moderate associations, the inclusion of accelerometry-derived features to predict clinical scores in the gold-standard clinical assessments for non-motor symptoms did not convincingly improve model performance beyond base models based on age, sex, and RBD symptom duration only. Whereas Schalkamp, Harrison [19] reported a poor predictive performance with no variance in clinical outcomes being explained through the smartwatch-derived digital features on physical activity and sleep, our models explained a significant amount of variance in the clinical outcomes. For global cognition, memory, visuo-cognition, as well as for overall non-motor symptom burden (NMSQ), fatigue (FSMC), and depressive symptoms (BDI-II), the accelerometry-derived features added, on average, more than 10% of explained variance to the base models. However, an inverse pattern was observed for clinical outcomes related to PD-related motor symptoms (MDS-UPDRS-III total score, and rigidity, axial, and bradykinesia subscores), and sleep disturbances (PDSS, RBDSQ), which were generally better predicted by age, sex, and RBD symptom duration than by the accelerometry-enriched feature set. Sensitivity analyses indicated potential differences in the prognostic value of daytime versus nighttime/circadian rhythm features for clinical outcomes. For example, the amount of explained variance by daytime versus nighttime/circadian rhythm features varied across different cognitive domains and motor subscores. Overall non-motor symptom burden (NMSQ), fatigue (FSMC), and depressive symptoms (BDI-II) were better predicted by nighttime/circadian rhythm features, potentially driven primarily by circadian rhythm-specific parameters.

Future studies should evaluate the longitudinal prognostic potential of accelerometry-derived features to predict clinical change in iRBD and should clarify whether the alterations of the digital measures found in this (and potentially also other) studies are associated with the disease, with the progression of the disease, with lifestyle habits, or are even constitutional factors. All these options justify further investigation in this field, and most probably strengthen the usefulness of collecting such digital measures, e.g., for treatment response assessment or as covariates that improve our understanding of prodromal PD / DLB symptoms. Furthermore, the sensitivity to change of these accelerometry-derived features should be investigated. As non-motor symptoms are known to be subject to fluctuations just as motor symptoms [53], digital measures in the prodromal phase preceding the clinical diagnosis of PD and DLB may be more sensitive as progression markers than traditional episodic in-clinic assessments [10]. Longitudinal data will also be crucial for gaining a better understanding of the bidirectional relationship between clinical outcomes and digital features. For example, depressive symptoms may lead to a more sedentary lifestyle, while a sedentary lifestyle may also contribute to the development of depressive symptoms. This highlights the possibility of reverse causation adding to the overall complexity.

### Strengths and limitations

Strengths of this study include the thorough characterization of our sample through both standardized clinical assessments and accelerometry, as well as the use of a readily available, standardized, open-source pipeline for accelerometry feature extraction [22], which will facilitate replication studies. One limitation of the study is the absence of a comprehensive sleep evaluation using polysomnography in the HC group, relying instead on the reported medical history for screening the HC. Moreover, participants were only asked to fill out daytime activity diaries; however, they did not complete sleep diaries or any measure regarding the severity of (RBD) sleep disturbances (e.g., RBD Questionnaire — Hong Kong, RBDQ-HK, [54]). Furthermore, potential comorbidities (e.g., asthma, chronic obstructive pulmonary disease) were not systematically recorded for HC. As a result, group comparisons, correlational analyses, and prediction models were not statistically controlled for comorbidities.

Methodologically, GGIR-implemented algorithms make several general assumptions that may not fully capture the unique sleep characteristics of individuals with iRBD. In particular, periods of evening inactivity may be misclassified as sleep onset, potentially leading to an overestimation of the SPT. Previous studies have demonstrated that GGIR-derived sleep parameters — based on the HDCZA and 5-min window / 5° angle threshold algorithm — tend to overestimate total sleep duration by an average of 31 min compared to polysomnography in healthy subjects [38]. Future studies comparing accelerometry-derived sleep metrics to polysomnography in iRBD populations are necessary to evaluate whether the current 5-min/5°threshold configuration is optimal or whether alternative parameter settings might improve accuracy for sleep detection in individuals with iRBD. Furthermore, using accelerometry alone, it is impossible to distinguish whether sleep interruptions are true wake periods or episodes of pathological motor activity during REM sleep in individuals with iRBD. This limitation may have introduced bias in the measurement of circadian rhythm and sleep parameters. However, any such bias would likely have resulted in more pronounced abnormalities in individuals with iRBD — such as higher WASO, lower sleep efficiency, lower SRI, lower IS, and higher IV — compared to HC, which we did not observe. Future accelerometry studies in iRBD may also evaluate more specific features capturing specific RBD activity patterns for predicting clinical outcomes in early α-synucleinopathies, for example, as described in Brink‐Kjaer, Gupta [17].

As to this point, no longitudinal data was available for analysis, we could not investigate the sensitivity of change and the longitudinal prognostic potential of the digital accelerometry-derived features of physical activity, sleep, and circadian rhythm within this project. Finally, as discussed above, the analyzed sample may be biased towards individuals with iRBD particularly pursuing a healthy, active lifestyle, making the group comparisons to HC less meaningful.

## Conclusion

Our findings contribute to the growing body of evidence highlighting the complex interplay between physical activity, sleep, circadian rhythm, and non-motor symptoms in α-synucleinopathies. While significant differences between iRBD and HC were not consistently observed, the relationships identified between accelerometry-derived measures and clinical outcomes provide valuable insights into potential targets for neuroprotective trials. Future research should focus on longitudinal studies to monitor changes in clinical outcomes and digital biomarkers over time to enhance our understanding of symptom progression and corresponding lifestyle changes in prodromal and manifest α-synucleinopathies.

## Supplementary Information

Below is the link to the electronic supplementary material.

Supplementary file1 (PDF 577 KB)

## Data Availability

The data supporting this study’s findings are available on reasonable request from the corresponding author AO. The data are not publicly available due to privacy or ethical restrictions.
