# Supplementary material for: Accelerometry-derived features of physical activity, sleep and circadian rhythm relate to non-motor symptoms in individuals with isolated REM sleep behavior disorder
Source: J Neurol. 2025 Feb 11;272(3):201. doi: 10.1007/s00415-025-12931-6 (PMC11813973; doi:10.1007/s00415-025-12931-6)
Supplement: Supplementary file 1 — Supplementary file1 (PDF 577 KB) [file 415_2025_12931_MOESM1_ESM.pdf]

ONLINE RESOURCES

Accelerometry-derived features of physical activity, sleep and circadian rhythm relate to non-motor symptoms in individuals with isolated REM sleep behavior disorder

Online Resource Table S1. Neuropsychological and motor assessments in CogTrAiL-RBD

Online Resource Table S2. GGIR-derived accelerometry features of interest

Online Resource Table S3. Further Clinical Characteristics and Differences in Physical Activity, Sleep, and Circadian Rhythm between iRBD and HC

Online Resource Table S4. Spearman's Rank Correlations between Physical Activity, Sleep, and Circadian Rhythm in iRBD

Online Resource Table S5. Pearson Correlations between Physical Activity, Sleep, and Circadian Rhythm in iRBD

Online Resource Table S6. Spearman's Rank Correlations of Physical Activity, Sleep, and Circadian Rhythm with Clinical Outcomes in iRBD

Online Resource Table S7. Pearson Correlations of Physical Activity, Sleep, and Circadian Rhythm with Clinical Outcomes in iRBD

Online Resource Table S8. Prediction of Clinical Scores from Accelerometry-Derived Measures: Comparison of Base Models (Covariates Only) and Accelerometry-Enriched Full Models

Online Resource S9. Prediction of Clinical Scores from Accelerometry-Derived Measures: Comparison of Base Models (Covariates Only) and Accelerometry-Enriched Full Models Differentiating Between Daytime and Nighttime Features

Online Resource S1. Neuropsychological and motor assessments in CogTrAiL-RBD

DOMAIN	Abbreviation	Assessment	Relevant for Level-II MCI Assessment	Reference
**COGNITION**				
**Overall Cognitive State**				
Subjective Cognition	Multi-SubCoDE	Multi-SubCoDE		used e.g. in Kalbe, Bintener [1]
Global Cognition	MoCA	Montreal Cognitive Assessment: Version B or A		Nasreddine, Phillips [2]
**Executive**				
Semantic fluency	RWT sem	Regensburger Wortflüssigkeitstest: Food or Animals	X*	Aschenbrenner, Tucha [3]
Phonemic fluency	RWT phon	Regensburger Wortflüssigkeitstest: P- or S-words	X*	Aschenbrenner, Tucha [3]
Set-Shifting	TMTB/A	Trail Making Test (TMT): TMT-B / TMT-A	X	Reitan [4]; Aebi [5]
Inhibition	Stroop-I	Stroop Interference		Bäumler and Stroop [6]
Logical Reasoning	LPS-4	Leistungsprüfsystem 50+: Subtest 4, Fluid Reasoning, Version A or B		Sturm, Willmes [7]
**Visuo-cognition**				
Construction	ROCFT	Rey Osterrieth Complex Figure Test (ROCFT): Figure Copy	X	Rey [8],Strauss, Sherman [9]
Perception	LPS-11	Leistungsprüfsystem 50+: Subtest 11, Visual Perception, Version A or B	X*	Sturm, Willmes [7]
Spatial Perception	BJLO	Benton Judgment of Line Orientation, Version V or H		Benton, Hannay [10],Benton [11]
	LPS-7	Leistungsprüfsystem 50+: Subtest 7, Spatial Rotation, Version A or B	X*	Sturm, Willmes [7]
**Attention & Working Memory**				
Working Memory	DSback	Wechsler Adult Intelligence Scale (WAIS): Digit Span backwards	X	Wechsler [12]
	BTA	Brief Test of Attention		Schretlen [13]
Processing Speed	TMT-A	Trail Making Test A	X	Reitan [4]; Aebi [5]
Attention	Stroop-W	Stroop Word Reading		Bäumler and Stroop [6]
	Stroop-C	Stroop Color Naming		Bäumler and Stroop [6]
**Memory**				
Verbal Memory	DSforw	Wechsler Adult Intelligence Scale (WAIS): Digit Span forwards		Wechsler [12]
	VLMT-Learn	Verbaler Lern- und Merkfähigkeitstest (VLMT): Wordlist Learning, Version C or A	X*	Helmstaedter and Durwen [14]
	VLMT-Rec	Verbaler Lern- und Merkfähigkeitstest (VLMT): Wordlist Recall, Version C or A	X*	Helmstaedter and Durwen [14]
Visuo-spatial Memory	ROCFT	Rey Osterrieth Complex Figure Test (ROCFT): Figure Recall	X	Rey [8],Strauss, Sherman [9]
**Language**				
Naming	ACL-Naming	Aphasia Check List, Subtest Naming	X	Kalbe, Reinhold [15]
Semantic and Abstraction	WAIS	Wechsler Adult Intelligence Scale (WAIS): Similarities	X	von Aster and Neubauer [16]

Notes. MCI was defined as a triad of (i) SCD as reported by the individual in the Multi-SubCoDE, (ii) presence of impaired test performance ≥1 Standard Deviation (SD, $z \leq -1$) below published normative data in at least two tests within one or more of the five cognitive domains (Level-II, specific tests are indicated with X*), and (iii) preserved functional independence [17].

Online Resource Table S2. Quality Control and GGIR-derived accelerometry features of interest

Variable		Definition			GGIR variable
QUALITY CONTROL					
			**Healthy Controls** *N* = 24	**iRBD** *N* = 68	
Number of 10 second epochs used as sphere data during auto-calibration			26457 (3777) [19773 - 34975]	26803 (4407) [4351 - 35897]	n.10sec.windows
Calibration error PRE		Calibration error prior to auto-calibration in m*g*	65 (56) [17 - 191]	41 (17) [14 - 81]	cal.error.start
Calibration error POST		Calibration error after auto-calibration in m*g*	4 (2) [2 - 10]	4 (1) [2 - 8]	cal.error.end
Clipping Score		Fraction of 15 minute windows per file for which the acceleration in one of the three axis was close to the maximum for at least 80% of the time. This should be 0.	0: 24 (100%)	0: 68 (100%)	clipping_score
Percentage of non-wear time		Non-wear percentage during the whole day, including waking and sleep period time	1.56 (2.32) [0 - 5.76]	0.64 (1.43) [0 - 6.66]	nonwear_perc_day_spt
PHYSICAL ACTIVITY					
**Total-ENMO**	Euclidean Norm Minus One gravitational unit	overall magnitude of acceleration in all three axes (X, Y and Z),calculated per day (0 - 24 h) averaged across all days	27.58 (8.69) [14.70 - 47.61]	26.86 (9.76) [12.77 - 71.39]	AD_mean_ENMO_mg_0-24hr
**IN**	Inactivity	Time during wake time spent in activities with minimal energy expenditure (i.e., ENMO 0–39 mg) in minutes; calculated per day (wake – sleep onset), averaged across days	664.60 (110.90) [463.33 - 860.00]	661.30 (92.86) [421.33 - 853.40]	dur_day_total_IN_min_pla
**LPA**	Light physical activity	Time during wake time spent in activities with light energy expenditure (i.e., ENMO 40–99 mg) in minutes; calculated per day (wake – sleep onset), averaged across days	218.59 (65.07) [82.83 - 328.00]	204.80 (50.88) [98.00 - 370.33]	dur_day_total_LIG_min_pla
**MPA**	Moderate physical activity	Time during wake time spent in activities with moderate energy expenditure (i.e., ENMO 100–399 mg) in minutes; calculated per day (wake – sleep onset), averaged across days	85.24 (47.40) [29.67 - 191.33]	83.42 (50.80) [5.17 - 226.67]	dur_day_total_MOD_min_pla
**VPA**	Vigorous physical activity	Time during wake time spent in activities with vigorous energy expenditure (i.e., ENMO ≥400 mg) in minutes; calculated per day (wake – sleep onset), averaged across days	3.58 (7.80) [0.00 - 35.00]	3.10 (7.87) [0.00 - 45.33]	dur_day_total_VIG_min_pla
SLEEP					
**WASO**	Wake After Sleep Onset	total time within the sleep period time (SPT) window estimated using the Heuristic algorithm looking at the Distribution of Change in Z-Angle (HDCZA) minus total time in minutes of sustained inactivity periods (SIP) within the SPT in hours; calculated per night (sleep onset – wake), averaged across nights	1.03 (0.47) [0.27 - 2.07]	1.35 (0.62) [0.38 - 2.85]	WASO_AD_T5A5_mn
**SleepEff**	sleep efficiency	percentage of SIP time within the SPT window; calculated per night (sleep onset – wake), averaged across nights	84.54 (8.18) [62.70 - 96.20]	83.00 (7.66) [62.30 - 95.70]	sleep_efficiency_after_onset_pla
CIRCADIAN RHYTHM					
**SRI**	Sleep Regularity Index	probability of an individual being in the same state (asleep vs. awake) at any two time points 24 h apart	55.83 (13.40) [30.77 - 78.76]	51.76 (9.69) [24.67 - 73.40]	SleepRegularityIndex_AD_T5A5_mn
**IS**	Interdaily Stability	between-day rhythm consistency with higher values indicating a more stable rhythm across days	0.62 (0.10) [0.45 - 0.78]	0.64 (0.10) [0.38 - 0.89]	IS_interdailystability
**IV**	Intradaily Variability	within-day fragmentation in the circadian rest-activity rhythm with lower values indicating a less fragmented rhythm	0.79 (0.21) [0.51 - 1.29]	0.72 (0.17) [0.33 - 1.15]	IV_intradailyvariability

Notes. Raw accelerometer data were preprocessed in R [18] with the open-source GGIR [19] R-package (version 3.1.4) to extract features on quality metrics, physical activity, sleep, and circadian rhythm. Data are mean (standard deviation) [Range: minimum-maximum] unless indicated otherwise.

Online Resource Table S3. Further Clinical Charcteristics and Differences in Physical Activity, Sleep, and Circadian Rhythm between iRBD and HC

		Healthy Controls *N* = 24	iRBD *N* = 68	*Mann-Whitney U Test*	p_{FDR}
**BDI-II category#**	**No depression**	21 (100%)	44 (68.8%)		
	**Minimal**	0 (0%)	11 (17.2%)		
	**Mild**	0 (0%)	7 (10.9%)		
	**Moderate**	0 (0%)	2 (3.1%)		
**Antidepressive medication**		0 (0%)	2 (2.9%) *Citalopram n = 1* *Moclobemid n = 1*		
**Dopamine agonists**		0 (0%)	0 (0%)		
**Constipation# (NMSQ Item 5)**		2 (9.5%)	25 (39.1%)		
**Orthostatic Hypotension# (NMSQ Item 20)**		1 (4.8%)	23 (35.9%)		
**SCOPA-AUT***		NA	8.13 (4.49) [0 - 19]		
**Daytime Sleepiness# (NMSQ Item 22)**		0 (0%)	5 (7.8%)		
**Restless Legs# (NMSQ Item 26)**		1 (4.8%)	4 (6.3%)		
**Respiratory medication***		NA	6 (8.8%)		
**Sniffin'Sticks***		NA	6.81 (2.67) [0 - 12] *normosmia n = 11* *hyposmia n = 36* *anosmia n = 15* *unknown n = 6*		
**Pathological DaTSCAN***		NA	15 (48.4%) *37 unknown*		
**Total-ENMO**		27.58 (8.69) [14.70 - 47.61]	26.86 (9.76) [12.77 - 71.39]	U = 893, p = .494, $\|r\|$ = 0.07	.617
**IN**		664.60 (110.90) [463.33 - 860.00]	661.30 (92.86) [421.33 - 853.40]	U = 809, p = .95, $\|r\|$ = 0.01	.950
**LPA**		218.59 (65.07) [82.83 - 328.00]	204.80 (50.88) [98.00 - 370.33]	U = 955, p = .216, $\|r\|$ = 0.13	.497
**MPA**		85.24 (47.40) [29.67 - 191.33]	83.42 (50.80) [5.17 - 226.67]	U = 829.5, p = .904, $\|r\|$ = 0.01	.950
**VPA**		3.58 (7.80) [0.00 - 35.00]	3.10 (7.87) [0.00 - 45.33]	U = 1011, p = .078, $\|r\|$ = 0.18	.370
**WASO**		1.03 (0.47) [0.27 - 2.07]	1.35 (0.62) [0.38 - 2.85]	U = 557, p = .021, $\|r\|$ = 0.24	.210
**SleepEff**		84.54 (8.18) [62.70 - 96.20]	83.00 (7.66) [62.30 - 95.70]	U = 933, p = .298, $\|r\|$ = 0.11	.497
**SRI**		55.83 (13.40) [30.77 - 78.76]	51.76 (9.69) [24.67 - 73.40]	U = 995, p = .111, $\|r\|$ = 0.17	.370
**IS**		0.62 (0.10) [0.45 - 0.78]	0.64 (0.10) [0.38 - 0.89]	U = 697, p = .29, $\|r\|$ = 0.11	.497
**IV**		0.79 (0.21) [0.51 - 1.29]	0.72 (0.17) [0.33 - 1.15]	U = 917, p = .369, $\|r\|$ = 0.09	.527

Notes. Data are mean (standard deviation) [Range] unless indicated otherwise. Healthy controls and individuals with iRBD were compared with Mann-Whitney U tests. BDI-II, Beck Depression Inventory; IN, time spent in inactivity; iRBD, isolated REM sleep behavior disorder; IS, interdaily stability; IV, intradaily variability; LPA, time spent in light physical activity; MPA, time spent in moderate physical activity; NMSQ, Non-Motor Symptom Questionnaire; SCOPA-AUT, Scales for Outcomes in Parkinson's Disease - Autonomic Dysfunction; SleepEff, sleep efficiency; SRI, Sleep Regularity Index; Total-ENMO, Euclidean norm minus one (ENMO) gravitational unit; VPA, time spent in vigorous physical activity; WASO, wake after sleep onset.

n_{HC} = 21, n_{RBD} = 64

*not assessed for the present trial, but during the annual clinical visits on average 105±207 days before the study visit; DaTSCAN 391±372 days before the study visit

Online Resource Table S4. Spearman's Rank Correlations between Physical Activity, Sleep, and Circadian Rhythm in iRBD

		Total-ENMO	IN	LPA	MPA	VPA	WASO	SleepEff	SRI	IS	IV
Total-ENMO	ρ	1	-0.696	0.525	0.893	0.505	0.032	-0.16	0.395	0.371	-0.42
	n		68	68	68	68	68	68	68	68	68
	p_{FDR}		< .001 ***	< .001 ***	< .001 ***	< .001 ***	0.83	0.253	0.002 **	0.004 **	0.001 **
IN	ρ	-0.696	1	-0.677	-0.72	-0.179	-0.186	0.21	-0.235	-0.493	0.541
	n	68		68	68	68	68	68	68	68	68
	p_{FDR}	< .001 ***		< .001 ***	< .001 ***	0.196	0.185	0.127	0.082 +	< .001 ***	< .001 ***
LPA	ρ	0.525	-0.677	1	0.421	-0.01	-0.016	-0.086	0.305	0.361	-0.586
	n	68	68		68	68	68	68	68	68	68
	p_{FDR}	< .001 ***	< .001 ***		0.001 **	0.936	0.915	0.579	0.02 *	0.005 **	< .001 ***
MPA	ρ	0.893	-0.72	0.421	1	0.407	0.129	-0.244	0.26	0.34	-0.329
	n	68	68	68		68	68	68	68	68	68
	p_{FDR}	< .001 ***	< .001 ***	0.001 **		0.001 **	0.376	0.07 +	0.052 +	0.008 **	0.011 *
VPA	ρ	0.505	-0.179	-0.01	0.407	1	-0.094	0.1	0.269	0.179	-0.072
	n	68	68	68	68		68	68	68	68	68
	p_{FDR}	< .001 ***	0.196	0.936	0.001 **		0.546	0.521	0.044 *	0.196	0.624
WASO	ρ	0.032	-0.186	-0.016	0.129	-0.094	1	-0.839	-0.518	-0.075	-0.044
	n	68	68	68	68	68		68	68	68	68
	p_{FDR}	0.83	0.185	0.915	0.376	0.546		< .001 ***	< .001 ***	0.623	0.784
SleepEff	ρ	-0.16	0.21	-0.086	-0.244	0.1	-0.839	1	0.391	0.036	0.074
	n	68	68	68	68	68	68		68	68	68
	p_{FDR}	0.253	0.127	0.579	0.07 +	0.521	< .001 ***		0.002 **	0.818	0.623
SRI	ρ	0.395	-0.235	0.305	0.26	0.269	-0.518	0.391	1	0.436	-0.4
	n	68	68	68	68	68	68	68		68	68
	p_{FDR}	0.002 **	0.082 +	0.02 *	0.052 +	0.044 *	< .001 ***	0.002 **		0.001 **	0.002 **
IS	ρ	0.371	-0.493	0.361	0.34	0.179	-0.075	0.036	0.436	1	-0.577
	n	68	68	68	68	68	68	68	68		68
	p_{FDR}	0.004 **	< .001 ***	0.005 **	0.008 **	0.196	0.623	0.818	0.001 **		< .001 ***
IV	ρ	-0.42	0.541	-0.586	-0.329	-0.072	-0.044	0.074	-0.4	-0.577	1
	n	68	68	68	68	68	68	68	68	68	
	p_{FDR}	0.001 **	< .001 ***	< .001 ***	0.011 *	0.624	0.784	0.623	0.002 **	< .001 ***	

Notes. The correlation matrix presents the partial Spearman's rank correlations adjusted for age. Reported is Spearman's ρ, the sample size, and the corresponding FDR-corrected significance levels. IN, time spent in inactivity; iRBD, isolated REM sleep behavior disorder; IS, interdaily stability; IV, intradaily variability; LPA, time spent in light physical activity; MPA, time spent in moderate physical activity; SleepEff, sleep efficiency; SRI, Sleep Regularity Index; Total-ENMO, Euclidean norm minus one (ENMO) gravitational unit; VPA, time spent in vigorous physical activity; WASO, wake after sleep onset.

*** $p_{FDR} < .001$, ** $p_{FDR} < .01$, * $p_{FDR} < .05$.

Online Resource Table S5. Pearson Correlations between Physical Activity, Sleep, and Circadian Rhythm in iRBD

		Total-ENMO	IN	LPA	MPA	VPA	WASO	SleepEff	SRI	IS	IV
Total-ENMO	*r*	1	-0.668	0.421	0.85	0.736	0.045	-0.194	0.311	0.483	-0.455
	n		68	68	68	68	68	68	68	68	68
	p_{FDR}		< .001 ***	0.001 **	< .001 ***	< .001 ***	0.776	0.164	0.017 *	< .001 ***	< .001 ***
IN	*r*	-0.668	1	-0.705	-0.752	-0.16	-0.177	0.193	-0.252	-0.577	0.593
	n	68		68	68	68	68	68	68	68	68
	p_{FDR}	< .001 ***		< .001 ***	< .001 ***	0.259	0.205	0.164	0.061 +	< .001 ***	< .001 ***
LPA	*r*	0.421	-0.705	1	0.418	-0.088	-0.055	-0.068	0.392	0.426	-0.657
	n	68	68		68	68	68	68	68	68	68
	p_{FDR}	0.001 **	< .001 ***		0.001 **	0.593	0.731	0.674	0.002 **	0.001 **	< .001 ***
MPA	*r*	0.85	-0.752	0.418	1	0.359	0.078	-0.23	0.251	0.439	-0.391
	n	68	68	68		68	68	68	68	68	68
	p_{FDR}	< .001 ***	< .001 ***	0.001 **		0.005 **	0.645	0.09 +	0.061 +	< .001 ***	0.002 **
VPA	ρ	0.736	-0.16	-0.088	0.359	1	-0.028	-0.034	0.142	0.257	-0.13
	n	68	68	68	68		68	68	68	68	68
	p_{FDR}	< .001 ***	0.259	0.593	0.005 **		0.853	0.835	0.327	0.057 +	0.374
WASO	*r*	0.045	-0.177	-0.055	0.078	-0.028	1	-0.817	-0.453	-0.013	-0.057
	n	68	68	68	68	68		68	68	68	68
	p_{FDR}	0.776	0.205	0.731	0.645	0.853		< .001 ***	< .001 ***	0.935	0.729
SleepEff	*r*	-0.194	0.193	-0.068	-0.23	-0.034	-0.817	1	0.32	0.01	0.074
	n	68	68	68	68	68	68		68	68	68
	p_{FDR}	0.164	0.164	0.674	0.09 +	0.835	< .001 ***		0.014 *	0.938	0.655
SRI	*r*	0.311	-0.252	0.392	0.251	0.142	-0.453	0.32	1	0.438	-0.477
	n	68	68	68	68	68	68	68		68	68
	p_{FDR}	0.017 *	0.061 +	0.002 **	0.061 +	0.327	< .001 ***	0.014 *		< .001 ***	< .001 ***
IS	*r*	0.483	-0.577	0.426	0.439	0.257	-0.013	0.01	0.438	1	-0.594
	n	68	68	68	68	68	68	68	68		68
	p_{FDR}	< .001 ***	< .001 ***	0.001 **	< .001 ***	0.057 +	0.935	0.938	< .001 ***		< .001 ***
IV	*r*	-0.455	0.593	-0.657	-0.391	-0.13	-0.057	0.074	-0.477	-0.594	1
	n	68	68	68	68	68	68	68	68	68	
	p_{FDR}	< .001 ***	< .001 ***	< .001 ***	0.002 **	0.374	0.729	0.655	< .001 ***	< .001 ***	

Notes. The correlation matrix presents the partial Pearson correlations adjusted for age. Reported is Pearson r, the sample size, and the corresponding FDR-corrected significance levels. IN, time spent in inactivity; iRBD, isolated REM sleep behavior disorder; IS, interdaily stability; IV, intradaily variability; LPA, time spent in light physical activity; MPA, time spent in moderate physical activity; SleepEff, sleep efficiency; SRI, Sleep Regularity Index; Total-ENMO, Euclidean norm minus one (ENMO) gravitational unit; VPA, time spent in vigorous physical activity; WASO, wake after sleep onset.

*** $p_{FDR} < .001$, ** $p_{FDR} < .01$, * $p_{FDR} < .05$.

Online Resource Table S6. Spearman's Rank Correlations of Physical Activity, Sleep, and Circadian Rhythm with Clinical Outcomes in iRBD

		MoCA	Global Cognition	Executive Functions	Attention & WM	Memory	Visuo-Cognition	Language	TUG Cost	Purdue PB	MDS-UPDRS-III	NMSQ	FSMC	BDI-II	PDSS	RBDSQ
Total-ENMO	ρ	0.017	0.054	-0.019	-0.033	0.041	0.023	0.076	-0.03	0.027	-0.043	-0.264	-0.32	-0.294	-0.035	0.011
	n	68	68	68	68	68	68	68	68	68	46	64	64	64	64	64
	p	0.892	0.662	0.877	0.787	0.738	0.855	0.537	0.809	0.829	0.779	0.035 *	0.01 *	0.018 *	0.783	0.932
	p_{FDR}	0.963	0.955	0.963	0.963	0.963	0.963	0.927	0.963	0.963	0.963	0.31	0.18	0.252	0.963	0.963
IN	ρ	0.092	-0.01	-0.017	-0.009	-0.007	0.012	0.069	-0.073	-0.26	-0.034	0.24	0.239	0.103	0.095	0.14
	n	68	68	68	68	68	68	68	68	68	46	64	64	64	64	64
	p	0.453	0.935	0.893	0.939	0.952	0.925	0.574	0.552	0.032 *	0.824	0.056 +	0.058 +	0.42	0.456	0.269
	p_{FDR}	0.927	0.963	0.963	0.963	0.964	0.963	0.927	0.927	0.303	0.963	0.375	0.375	0.927	0.927	0.859
LPA	ρ	0.079	0.127	0.009	0.097	0.164	0.026	0.052	0.186	0.11	0.011	-0.099	-0.153	-0.19	-0.029	0.074
	n	68	68	68	68	68	68	68	68	68	46	64	64	64	64	64
	p	0.521	0.302	0.944	0.431	0.181	0.833	0.672	0.129	0.37	0.944	0.435	0.228	0.133	0.822	0.561
	p_{FDR}	0.927	0.906	0.963	0.927	0.716	0.963	0.96	0.687	0.911	0.963	0.927	0.814	0.687	0.963	0.927
MPA	ρ	0.026	0.11	0.061	-0.013	0.081	0.092	0.091	-0.043	0.178	-0.083	-0.372	-0.369	-0.312	-0.174	-0.035
	n	68	68	68	68	68	68	68	68	68	46	64	64	64	64	64
	p	0.834	0.37	0.622	0.915	0.51	0.454	0.462	0.727	0.147	0.585	0.002 **	0.003 **	0.012 *	0.169	0.785
	p_{FDR}	0.963	0.911	0.951	0.963	0.927	0.927	0.927	0.963	0.687	0.933	0.081 +	0.081 +	0.182	0.705	0.963
VPA	ρ	0.023	0.034	0.055	-0.029	-0.116	0.03	0.064	0.025	0.009	-0.249	-0.011	-0.18	-0.106	0.148	0.117
	n	68	68	68	68	68	68	68	68	68	46	64	64	64	64	64
	p	0.853	0.786	0.655	0.815	0.344	0.807	0.602	0.841	0.941	0.096 +	0.929	0.155	0.404	0.244	0.356
	p_{FDR}	0.963	0.963	0.955	0.963	0.911	0.963	0.941	0.963	0.963	0.573	0.963	0.69	0.927	0.814	0.911
WASO	ρ	-0.076	-0.166	-0.097	-0.112	-0.117	-0.172	-0.253	-0.323	0.045	-0.188	-0.027	-0.075	0.155	0.056	0.245
	n	68	68	68	68	68	68	68	68	68	46	64	64	64	64	64
	p	0.536	0.176	0.433	0.363	0.343	0.161	0.038 *	0.007 **	0.714	0.211	0.835	0.553	0.223	0.658	0.051 +
	p_{FDR}	0.927	0.714	0.927	0.911	0.911	0.69	0.313	0.178	0.963	0.811	0.963	0.927	0.814	0.955	0.375
SleepEff	ρ	0.057	0.085	0.054	0.016	0.045	0.146	0.24	0.28	-0.005	0.131	0.073	0.199	0.012	-0.131	-0.204
	n	68	68	68	68	68	68	68	68	68	46	64	64	64	64	64
	p	0.642	0.49	0.662	0.899	0.718	0.236	0.049 *	0.021 *	0.971	0.386	0.565	0.114	0.925	0.301	0.105
	p_{FDR}	0.955	0.927	0.955	0.963	0.963	0.814	0.375	0.258	0.971	0.927	0.927	0.634	0.963	0.906	0.607
SRI	ρ	0.178	0.082	0.093	0.075	0.047	0.075	0.074	0.179	0.045	0.016	-0.235	-0.269	-0.325	0.062	-0.119
	n	68	68	68	68	68	68	68	68	68	46	64	64	64	64	64
	p	0.146	0.506	0.451	0.545	0.704	0.542	0.547	0.143	0.714	0.914	0.062 +	0.032 *	0.009 **	0.628	0.349
	p_{FDR}	0.687	0.927	0.927	0.927	0.963	0.927	0.927	0.687	0.963	0.963	0.387	0.303	0.18	0.952	0.911
IS	ρ	-0.087	-0.065	-0.03	-0.028	-0.024	-0.12	-0.146	0.112	0.132	-0.097	-0.317	-0.419	-0.282	-0.241	-0.043
	n	68	68	68	68	68	68	68	68	68	46	64	64	64	64	64
	p	0.483	0.598	0.807	0.819	0.847	0.331	0.234	0.364	0.282	0.521	0.011 *	0.001 **	0.024 *	0.055 +	0.739
	p_{FDR}	0.927	0.941	0.963	0.963	0.963	0.911	0.814	0.911	0.88	0.927	0.18	0.045 *	0.278	0.375	0.963
IV	ρ	-0.069	-0.095	-0.094	-0.116	0.007	-0.172	0.145	-0.138	-0.118	-0.106	0.268	0.454	0.389	0.076	-0.064
	n	68	68	68	68	68	68	68	68	68	46	64	64	64	64	64
	p	0.575	0.443	0.445	0.346	0.958	0.161	0.24	0.262	0.337	0.484	0.032 *	< .001 ***	0.002 **	0.55	0.617
	p_{FDR}	0.927	0.927	0.927	0.911	0.964	0.69	0.814	0.853	0.911	0.927	0.303	0.03 *	0.075 +	0.927	0.951

Notes. The correlation matrix reports the partial Spearman's rank correlation coefficients (ρ) adjusted for age, sample size, uncorrected and FDR-corrected significance level. BDI-II, Beck Depression Inventory; FSMC, Fatigue Scale for Motor and Cognitive Functions; IN, time spent in inactivity; iRBD, isolated REM sleep behavior disorder; IS, interdaily stability; IV, intradaily variability; LPA, time spent in light physical activity; MDS-UPDRS-III, Movement Disorder Society Unified Parkinson's Disease Rating Scale Part 3; MoCA, Montréal Cognitive Assessment; MPA, time spent in moderate physical activity; NMSQ, Non-Motor Symptom Questionnaire; PDSS, Parkinson's Disease Sleep Scale; Purdue PB, Purdue Pegboard dominant hand; RBDSQ, REM Sleep Behavior Disorder Screening Questionnaire; SleepEff, sleep efficiency; SRI, Sleep Regularity Index; Total-ENMO, Euclidean norm minus one (ENMO) gravitational unit; TUG Cost, Timed Up and Go Cost single task – dual task; VPA, time spent in vigorous physical activity; WASO, wake after sleep onset.

*** $p_{FDR} < .001$, ** $p_{FDR} < .01$, * $p_{FDR} < .05$, + $p_{FDR} < .10$.

Online Resource Table S7. Pearson Correlations of Physical Activity, Sleep, and Circadian Rhythm with Clinical Outcomes in iRBD

		**MoCA**	**Global Cognition**	**Executive Functions**	**Attention & WM**	**Memory**	**Visuo-Cognition**	**Language**	**TUG Cost**	**Purdue PB**	**MDS-UPDRS-III**	**NMSQ**	**FSMC**	**BDI-II**	**PDSS**	**RBDSQ**
Total-ENMO	*r*	-0.107	-0.082	-0.14	-0.103	-0.002	-0.038	0.005	-0.026	0.008	0.012	-0.245	-0.329	-0.229	0.002	0.075
	n	68	68	68	68	68	68	68	68	68	46	64	64	64	64	64
	p	0.384	0.508	0.254	0.404	0.987	0.76	0.971	0.832	0.951	0.935	0.051 +	0.008 **	0.068 +	0.987	0.555
	p_{FDR}	0.86	0.867	0.809	0.86	0.987	0.975	0.987	0.975	0.987	0.987	0.413	0.145	0.413	0.987	0.874
IN	*r*	0.073	-0.002	0.022	0.01	-0.055	0.017	0.022	-0.085	-0.233	-0.032	0.296	0.279	0.227	0.151	0.029
	n	68	68	68	68	68	68	68	68	68	46	64	64	64	64	64
	p	0.552	0.986	0.858	0.935	0.656	0.887	0.856	0.492	0.056 +	0.835	0.018 *	0.025 *	0.071 +	0.234	0.821
	p_{FDR}	0.874	0.987	0.975	0.987	0.947	0.987	0.975	0.86	0.413	0.975	0.203	0.254	0.413	0.762	0.975
LPA	*r*	0.125	0.137	0.005	0.094	0.209	0.035	0.103	0.206	0.089	0.07	-0.167	-0.223	-0.288	-0.065	0.074
	n	68	68	68	68	68	68	68	68	68	46	64	64	64	64	64
	p	0.311	0.266	0.966	0.447	0.087 +	0.779	0.403	0.092 +	0.469	0.642	0.187	0.076 +	0.021 *	0.61	0.559
	p_{FDR}	0.84	0.831	0.987	0.86	0.433	0.975	0.86	0.438	0.86	0.946	0.667	0.413	0.225	0.92	0.874
MPA	*r*	-0.023	0.026	-0.025	-0.072	0.088	0.037	0.087	-0.05	0.194	-0.019	-0.412	-0.395	-0.355	-0.194	-0.013
	n	68	68	68	68	68	68	68	68	68	46	64	64	64	64	64
	p	0.849	0.834	0.841	0.557	0.477	0.764	0.48	0.684	0.113	0.9	0.001 **	0.001 **	0.004 **	0.125	0.92
	p_{FDR}	0.975	0.975	0.975	0.874	0.86	0.975	0.86	0.975	0.515	0.987	0.052 +	0.06 +	0.114	0.548	0.987
VPA	*r*	-0.178	-0.16	-0.147	-0.103	-0.117	-0.118	-0.079	-0.015	-0.163	0.031	0.014	-0.109	0.002	0.135	0.099
	n	68	68	68	68	68	68	68	68	68	46	64	64	64	64	64
	p	0.147	0.194	0.231	0.401	0.341	0.337	0.522	0.905	0.185	0.838	0.91	0.391	0.985	0.289	0.438
	p_{FDR}	0.567	0.675	0.762	0.86	0.84	0.84	0.87	0.987	0.667	0.975	0.987	0.86	0.987	0.84	0.86
WASO	*r*	-0.043	-0.225	-0.12	-0.123	-0.181	-0.182	-0.228	-0.294	0.062	-0.157	-0.035	-0.045	0.103	0.072	0.236
	n	68	68	68	68	68	68	68	68	68	46	64	64	64	64	64
	p	0.726	0.065 +	0.331	0.319	0.139	0.138	0.062 +	0.015 *	0.614	0.298	0.786	0.723	0.416	0.572	0.061 +
	p_{FDR}	0.975	0.413	0.84	0.84	0.558	0.558	0.413	0.186	0.92	0.84	0.975	0.975	0.86	0.875	0.413
SleepEff	*r*	0.023	0.118	0.045	0.024	0.073	0.124	0.225	0.216	0.009	0.125	0.093	0.219	0.058	-0.137	-0.249
	n	68	68	68	68	68	68	68	68	68	46	64	64	64	64	64
	p	0.85	0.336	0.717	0.844	0.555	0.312	0.066 +	0.077 +	0.94	0.408	0.465	0.081 +	0.652	0.28	0.048 *
	p_{FDR}	0.975	0.84	0.975	0.975	0.874	0.84	0.413	0.413	0.987	0.86	0.86	0.421	0.947	0.84	0.413
SRI	*r*	0.18	0.092	0.086	0.048	0.048	0.12	0.041	0.187	0.012	-0.035	-0.178	-0.311	-0.262	0.091	-0.098
	n	68	68	68	68	68	68	68	68	68	46	64	64	64	64	64
	p	0.141	0.456	0.487	0.695	0.697	0.331	0.739	0.128	0.925	0.815	0.159	0.013 *	0.036 *	0.476	0.442
	p_{FDR}	0.558	0.86	0.86	0.975	0.975	0.84	0.975	0.548	0.987	0.975	0.597	0.17	0.34	0.86	0.86
IS	*r*	-0.106	-0.043	0.003	-0.024	0.03	-0.079	-0.152	0.028	0.09	-0.131	-0.325	-0.467	-0.344	-0.226	-0.02
	n	68	68	68	68	68	68	68	68	68	46	64	64	64	64	64
	p	0.389	0.726	0.983	0.849	0.806	0.521	0.216	0.823	0.466	0.387	0.009 **	< .001 ***	0.005 **	0.072 +	0.876
	p_{FDR}	0.86	0.975	0.987	0.975	0.975	0.87	0.738	0.975	0.86	0.86	0.145	0.015 *	0.114	0.413	0.987
IV	*r*	-0.086	-0.082	-0.035	-0.071	-0.057	-0.205	0.088	-0.125	-0.118	-0.104	0.313	0.386	0.345	0.099	-0.08
	n	68	68	68	68	68	68	68	68	68	46	64	64	64	64	64
	p	0.487	0.504	0.777	0.566	0.643	0.094 +	0.478	0.31	0.34	0.493	0.012 *	0.002 **	0.005 **	0.438	0.532
	p_{FDR}	0.86	0.867	0.975	0.875	0.946	0.438	0.86	0.84	0.84	0.86	0.17	0.06 +	0.114	0.86	0.874

Notes. The correlation matrix reports the partial Pearson correlation coefficients (*r*) adjusted for age, sample size, uncorrected and FDR-corrected significance level. BDI-II, Beck Depression Inventory; FSMC, Fatigue Scale for Motor and Cognitive Functions; IN, time spent in inactivity; iRBD, isolated REM sleep behavior disorder; IS, interdaily stability; IV, intradaily variability; LPA, time spent in light physical activity; MDS-UPDRS-III, Movement Disorder Society Unified Parkinson's Disease Rating Scale Part 3; MoCA, Montréal Cognitive Assessment; MPA, time spent in moderate physical activity; NMSQ, Non-Motor Symptom Questionnaire; PDSS, Parkinson's Disease Sleep Scale; Purdue PB, Purdue Pegboard dominant hand; RBDSQ, REM Sleep Behavior Disorder Screening Questionnaire; SleepEff, sleep efficiency; SRI, Sleep Regularity Index; Total-ENMO, Euclidean norm minus one (ENMO) gravitational unit; TUG Cost, Timed Up and Go Cost single task – dual task; VPA, time spent in vigorous physical activity; WASO, wake after sleep onset.

*** $p_{FDR} < .001$, ** $p_{FDR} < .01$, * $p_{FDR} < .05$, + $p_{FDR} < .10$.

Online Resource Table S8. Prediction of Clinical Scores from Accelerometry-Derived Measures: Comparison of Base Models (Covariates Only) and Accelerometry-Enriched Full Models

	Base Model		Full Model					
	mean R^2	SD R^2	mean R^2	SD R^2	*t*	df	*p*	Cohen's *d*
MoCA	0.16	0.22	0.22	0.29	-0.56	18	.582	-0.25
Global Cognition	0.09	0.08	0.22	0.16	-2.23	18	.039	-1.00
Executive Functions	0.21	0.21	0.19	0.18	0.24	18	.817	0.11
Attention & Working Memory	0.14	0.16	0.15	0.17	-0.06	18	.951	-0.03
Memory	0.08	0.1	0.24	0.21	-2.21	18	.040	-0.99
Visuo-Cognition	0.14	0.15	0.24	0.27	-1.06	18	.301	-0.48
Language	0.26	0.21	0.24	0.27	0.19	18	.851	0.09
Timed Up and Go Cost, in seconds	0.26	0.27	0.21	0.24	0.45	18	.655	0.20
Purdue Pegboard, dominant hand	0.09	0.11	0.14	0.13	-1.05	18	.308	-0.47
MDS-UPDRS-III	0.35	0.28	0.2	0.25	1.3	18	.211	0.58
… rigidity	0.36	0.31	0.33	0.34	0.2	18	0.85	0.09
… axial	0.24	0.27	0.19	0.17	0.55	18	0.59	0.24
… bradykinesia	0.3	0.39	0.23	0.16	0.48	18	0.64	0.22
NMSQ	0.17	0.28	0.27	0.28	-0.79	18	.438	-0.35
FSMC	0.18	0.18	0.3	0.21	-1.4	18	.179	-0.62
BDI-II	0.14	0.12	0.26	0.3	-1.23	18	.234	-0.55
PDSS	0.31	0.3	0.18	0.22	1.1	18	.286	0.49
RBDSQ	0.19	0.19	0.12	0.12	1.03	18	.316	0.46

Notes. Two models per clinical outcome were compared. A regression models using the digital features on physical activity, sleep, and circadian rhythm as predictors, with age, sex, and time since first RBD symptoms as covariates was built as the 'full model'. For each clinical score, this full model was compared to a model based on covariates only ('base model'). Predictors and covariates were mean-centered prior to model estimation. All models employed elastic net regularization and were fitted using a nested ten-fold cross-validation. Model performance was reported as the mean and standard deviation of the R^2 scores across the ten outer folds and compared between base and full models with two-sided independent sample *t*-tests.

BDI-II, Beck Depression Inventory; FSMC, Fatigue Scale for Motor and Cognitive Functions; iRBD, isolated REM sleep behavior disorder; MCI, mild cognitive impairment; MDS-UPDRS-III, Movement Disorder Society Unified Parkinson's Disease Rating Scale Part 3; MoCA, Montréal Cognitive Assessment; NMSQ, Non-Motor Symptom Questionnaire; PDSS, Parkinson's Disease Sleep Scale; RBDSQ, REM Sleep Behavior Disorder Screening Questionnaire

Online Resource S9. Prediction of Clinical Scores from Accelerometry-Derived Measures: Comparison of Base Models (Covariates Only) and Accelerometry-Enriched Full Models Differentiating Between Daytime and Nighttime Features

To compare the contribution of daytime and nighttime accelerometry-derived features, we build to additional models per clinical outcome: Full Model DAY and Full Model NIGHT. In addition to the covariates, the Full Model DAY contains physical activity features, i.e., Total-ENMO, IN, LPA, MPA, and VPA. In addition to the covariates, the Full Model NIGHT contains nighttime and circadian rhythm features, i.e., WASO, SleepEff, SRI, IS, and IV. For a description of features, see Online Resource Table S2. Note, that due to a different, non-nested, set of features between the Full Model DAY and the Full Model NIGHT, the two are not directly comparable.

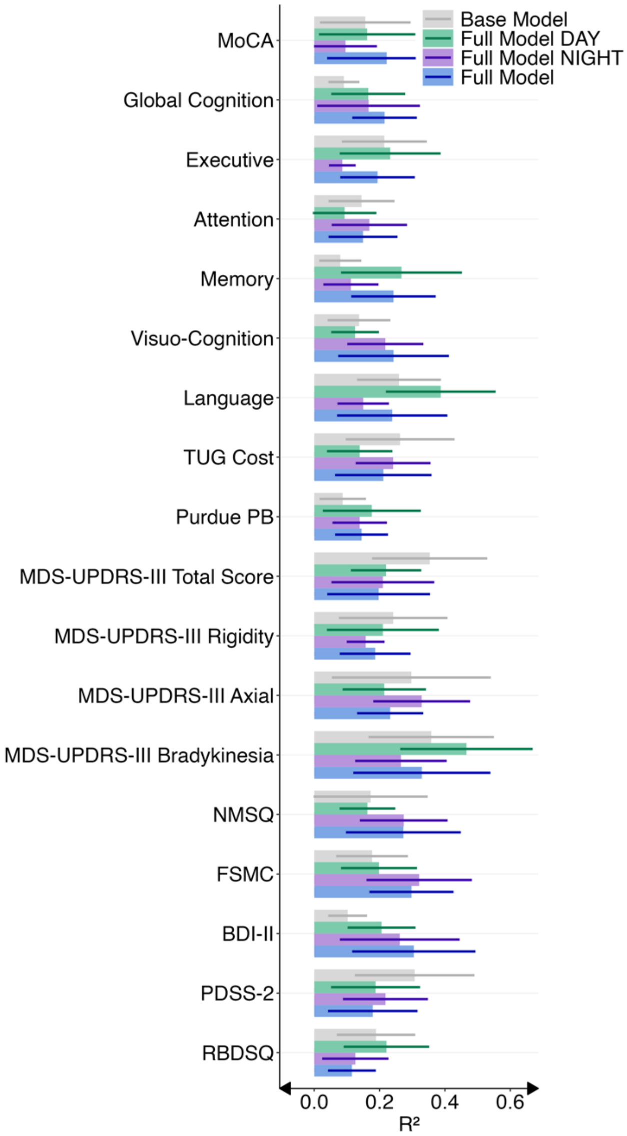

The amount of explained variance (R^2, x-axis) for each clinical measure (y-axis) is plotted as the mean R^2 across the ten outer cross-validation folds with its 95 % confidence interval for each model. BDI-II, Beck Depression Inventory; FSMC, Fatigue Scale for Motor and Cognitive Functions; iRBD, isolated REM sleep behavior disorder; MDS-UPDRS-III, Movement Disorder Society Unified Parkinson's Disease Rating Scale Part 3; MoCA, Montréal Cognitive Assessment; NMSQ, Non-Motor Symptom Questionnaire; PDSS, Parkinson's Disease Sleep Scale; Purdue PB, Purdue Pegboard dominant hand; RBDSQ, REM Sleep Behavior Disorder Screening Questionnaire; TUG Cost, Timed Up and Go Cost single task – dual task.

References

1. Kalbe, E., et al., *Computerized cognitive training in healthy older adults: Baseline cognitive level and subjective cognitive concerns predict training outcome.* Health, 2018. **10**(1): p. 20-55.
2. Nasreddine, Z.S., et al., *The Montreal Cognitive Assessment, MoCA: A brief screening tool for mild cognitive impairment.* Journal of the American Geriatrics Society, 2005. **53**(4): p. 695-699.
3. Aschenbrenner, S., O. Tucha, and K. Lange, *Regensburger Wortflüssigkeitstest Hogrefe Göttingen*. 2000, Hogrefe: Göttingen, Germany.
4. Reitan, R., *Trail Making Test: Manual for administration and scoring.* . 1992, Tucson, Arizona: Reitan Neuropsychology Laboratory.
5. Aebi, C., *Validierung der neuropsychologischen Testbatterie CERAD-NP: eine Multi-Center Studie*. 2002, University of Basel: Basel.
6. Bäumler, G. and J. Stroop, *Farbe-Wort-Interferenztest nach JR Stroop (FWIT)*. 1985: Hogrefe, Verlag für Psychologie.
7. Sturm, W., K. Willmes, and W. Horn, *Leistungsprüfsystem für 50–90jährige. Handanweisung*. 1993, Göttingen: Hogrefe.
8. Rey, A., *L'examen psychologique dans les cas d'encéphalopathie traumatique.(Les problems.).* Archives de psychologie, 1941.
9. Strauss, E., E.M. Sherman, and O. Spreen, *A compendium of neuropsychological tests: Administration, norms, and commentary*. 2006: American chemical society.
10. Benton, A., H.J. Hannay, and N.R. Varney, *Visual perception of line direction in patients with unilateral brain disease.* Neurology, 1975. **25**(10): p. 907-907.
11. Benton, A.L., *Contributions to neuropsychological assessment: A clinical manual*. 1994: Oxford University Press, USA.
12. Wechsler, D., *WMS-R: Wechsler memory scale-revised: Manual*. 1984: Psychological Corporation.
13. Schretlen, D., *Brief test of attention*. 1989, Baltimore: Psychological Assessment Resources.
14. Helmstaedter, C. and H. Durwen, *VLMT: Verbaler Lern-und Merkfähigkeitstest: Ein praktikables und differenziertes Instrumentarium zur Prüfung der verbalen Gedächtnisleistungen.* Schweizer Archiv für Neurologie, Neurochirurgie und Psychiatrie, 1990.
15. Kalbe, E., et al., *Aphasie-Check-Liste (ACL): Protokollheft, Testheft, Lösungsfolien, Vorlagen, Manual*. 2002, Köln: ProLog, Therapie-und Lernmittel.
16. von Aster, M. and A. Neubauer, *Wechsler-intelligenztest für erwachsene: WIE; manual; übersetzung und adaptation der WAIS-III von David Wechsler*. 2009: Pearson Assessment & Information.
17. Litvan, I., et al., *Diagnostic criteria for mild cognitive impairment in Parkinson's Disease: Movement Disorder Society Task Force guidelines.* Movement Disorders, 2012. **27**(3): p. 349-356.
18. R Core Team, *R: A language and environment for statistical computing. R Foundation for Statistical Computing, Vienna, Austria. URL https://www.R-project.org/*. 2023: Vienna, Austria.
19. Migueles, J.H., et al., *GGIR: A Research Community–Driven Open Source R Package for Generating Physical Activity and Sleep Outcomes From Multi-Day Raw Accelerometer Data.* Journal for the Measurement of Physical Behaviour, 2019. **2**(3): p. 188-196.
